# Enhancing ReaxFF for molecular dynamics simulations of lithium-ion batteries: an interactive reparameterization protocol

**DOI:** 10.1038/s41598-023-50978-5

**Published:** 2024-01-10

**Authors:** Paolo De Angelis, Roberta Cappabianca, Matteo Fasano, Pietro Asinari, Eliodoro Chiavazzo

**Affiliations:** 1 Department of Energy “Galileo Ferraris”, Politecnico di Torino, Corso Duca degli Abruzzi 24, 10129 Torino, Italy; 2 Istituto Nazionale di Ricerca Metrologica, Strada delle Cacce 91, 10135 Torino, Italy

**Keywords:** Batteries, Computational science, Molecular dynamics

## Abstract

Lithium-ion batteries (LIBs) have become an essential technology for the green economy transition, as they are widely used in portable electronics, electric vehicles, and renewable energy systems. The solid-electrolyte interphase (SEI) is a key component for the correct operation, performance, and safety of LIBs. The SEI arises from the initial thermal metastability of the anode-electrolyte interface, and the resulting electrolyte reduction products stabilize the interface by forming an electrochemical buffer window. This article aims to make a first—but important—step towards enhancing the parametrization of a widely-used reactive force field (ReaxFF) for accurate molecular dynamics (MD) simulations of SEI components in LIBs. To this end, we focus on Lithium Fluoride (LiF), an inorganic salt of great interest due to its beneficial properties in the passivation layer. The protocol relies heavily on various Python libraries designed to work with atomistic simulations allowing robust automation of all the reparameterization steps. The proposed set of configurations, and the resulting dataset, allow the new ReaxFF to recover the solid nature of the inorganic salt and improve the mass transport properties prediction from MD simulation. The optimized ReaxFF surpasses the previously available force field by accurately adjusting the diffusivity of lithium in the solid lattice, resulting in a two-order-of-magnitude improvement in its prediction at room temperature. However, our comprehensive investigation of the simulation shows the strong sensitivity of the ReaxFF to the training set, making its ability to interpolate the potential energy surface challenging. Consequently, the current formulation of ReaxFF can be effectively employed to model specific and well-defined phenomena by utilizing the proposed interactive reparameterization protocol to construct the dataset. Overall, this work represents a significant initial step towards refining ReaxFF for precise reactive MD simulations, shedding light on the challenges and limitations of ReaxFF force field parametrization. The demonstrated limitations emphasize the potential for developing more versatile and advanced force fields to upscale ab initio simulation through our interactive reparameterization protocol, enabling more accurate and comprehensive MD simulations in the future.

## Introduction

Lithium-ion batteries (LIBs) are and will continue to play an important role in the energy transition for energy storage and the fossil fuel replacement^1–4^. In the last decades, the growing popularity of electric vehicles, and energy storage systems^5^ has fuelled the demand for batteries with greater capacity, more consistent performance over time, and improved safety^6^. While the materials used as anodes and cathodes largely determine battery capacity, the degradation phenomena that occur within the device govern battery stability and safety. One of the most important and yet poorly understood of these phenomena is the formation of a thin passivization film at the electrode-electrolyte interface, known as the solid electrolyte interphase (SEI)^7–9^. This layer is formed by the irreversible reaction of lithium ions with the electrolyte due to the initial thermodynamic instability between the anode and electrolyte^10,11^. The ions used to create the SEI are subtracted from the battery’s capacity. Indeed the increase in the SEI over time is one of the causes of capacity fade observed during battery charging cycles^12^. Additionally, the uncontrolled formation and growth of the SEI layer can limit the performance and life of current LIBs, and its degradation can lead to severe battery damage and uncontrolled exothermic reactions such as the battery thermal runaway^13^. Therefore, it is essential to model the SEI to understand and predict the behaviour of lithium-ion batteries and improve their performance and safety^14^. Despite the importance of SEI, it remains a conundrum due to its high reactivity and multiscale nature, which make challenging its study *in operando* and *in silico*, respectively^3,15,16^. The theoretical understanding of the SEI may allow control of its final characteristics and it can enable the engineering of such layer, thus possibly leading to enhanced properties including better electronic insulation, prevention or alleviation of graphite anode exfoliation^17^ and dendrite formation in lithium metal batteries^18^. In addition, the modelling and subsequent theoretical understanding of SEI can contribute to significantly accelerate the discovery of new materials and electrolytes for future lithium-ion batteries^3,16^.

In the past two decades, various atomistic techniques have been utilized to study the kinetics of electrolyte dissociation in lithium-ion batteries^14^. The density functional theory (DFT) method has proven particularly useful in predicting the effects of lithium salt and additives on the final components of the solid electrolyte interphase^19–24^. Additionally, DFT has been used to investigate how the surface of the anode affects the electrolyte dissociation^25–28^ and to calculate various properties of SEI products, including ionic conductivity^29–31^, electron-transfer properties^28,32,33^, and elastic modulus^34–36^. Although such simulations produce highly accurate results, they are computationally expensive. The cost of DFT simulations increases with the number of atoms in the system, typically scaling with $O(N^2)$ where *N* is the number of atoms^37^, limiting the size of the system that can be simulated to a few thousand atoms and the time interval to a few picoseconds. To observe the evolution of the SEI over extended periods, one solution is to use approximate functions $V\left( \textbf{r}_i\right)$, known as force fields (FF), to calculate the system’s energy. Indeed, the physical intuition-based functions employed in many force fields enable the replacement of the expensive Kohn-Sham functional $E\left[ n\right]$^38^, based on electronic density *n*, with an approximate functional $E\left[ V\left( \textbf{r}_i\right) \right]$ that depends on the atomic configuration of the system $\textbf{r}_i$ neglecting the degree of freedom of the electrons and thus being computationally less demanding^38^. Given the reactive nature of lithium-ion batteries, traditional FF methods, which rely on harmonic laws to approximate bonded interactions, are inadequate for their study. To overcome this limitation, the ReaxFF method^39^, which utilizes the bond-order (BO) and electron equilibration method (EEM)^40^ to describe the connectivity and charges of atoms, respectively, has been suggested and utilized. This enabled the use of molecular dynamics (MD) simulations to observe the electrolyte decomposition and the formation of SEI components in LIBs. For instance, Kim et al.^41^ used ReaxFF optimized for C–H–O–Li species by Han at al.^42^ to study the formation of the SEI on lithium metal surfaces. They observed the production and layering of organic and inorganic components in the SEI on lithium metal surface, resulting from the decomposition of an Ethylene carbonate (EC) and Dimethyl carbonate (DMC) electrolyte mixture. Using the same force field, Guk et al.^43^ demonstrated how the layer of SEI that forms on the surface of graphite prevents the percolation of the electrolyte and the anode exfoliation. After this initial work, Yun et al.^44^ expanded the FF to include C-H-O-Li-Si-Li-F atoms, enabling the study of SEI formation in high-capacity batteries where graphite is replaced with silicon, which has a higher theoretical capacity ($372\,\hbox {mA}\,\hbox {h}\,\hbox {g}^{-1}$ for $\textrm{LiC}_{6}$ and $4212\,\hbox {mA}\,\hbox {h}\,\hbox {g}^{-1}$ for $\textrm{Li}_{4.4}\textrm{Si}$)^45^. This parameterization was then further enhanced by Wang et al.^46^ Current available ReaxFFs have demonstrated success in reproducing dissociation energies and reaction kinetics. However, as discussed below in this work, they fall short in accurately describing the solid phase of the SEI components.

In this study, we propose a protocol for reparameterizing the Yun et al. ReaxFF for C-H-O-Li-Si-Li-F atoms. Our approach focuses on correcting parameters related to Li and F atoms to better capture the properties of lithium fluoride (LiF), which is one of the possible inorganic salts resulting from the formation of SEI. LiF stems mainly from fluoroethylene carbonate (FEC) decomposition: LiF-rich SEI is known to exhibit improved cycling stability in batteries^47^. Furthermore, LiF has exceptional properties such as high ion transport, electronic insulation, and mechanical properties^48^, making it a crucial element in the engineering of the solid electrolyte interface, as demonstrated by Tan et al.^49^ While addressing a computational challenge, this work is also a critical step towards the broader and long-term goal of designing a multi-scale modelling framework for LIBs, which, together with additional experimental efforts, can help significantly to close some of the current technological gaps^3^. These methods are instrumental in comprehensively understanding the complex dynamics and energetics in LIB materials. This includes insights into electron localization in cathode materials, lithium diffusion dynamics, and the prediction of SEI formation. In this framework, computational innovation is key to integrating various scales and corresponding modelling methods. This integration aims to predict LIB performance from the material level up to the full battery pack design. For a deeper understanding of these challenges, we recommend further reading the works of Shi et al.^50^, Wang et al.^14^, and Cappabianca et al.^9^, which provide valuable insights into the multi-scale computational approach in LIB research.

The advancements in powerful python libraries for the management of atomistic systems, as outlined in the Table 1, have enabled the automation and orchestration of atomistic simulations using Jupyter notebooks and libraries such as ASE^51,52^, PyMatgen^53,54^, and ParAMS^55,56^. These tools have facilitated the parametrization process and made it more efficient. By sharing the Jupyter notebooks^57,58^ and the database used in this parametrization work, Authors hope to address a current gap in the field of ReaxFF parameterization, i.e., the lack of access to raw data and datasets, which can limit the reproducibility and validity of the results presented.

This article is structured as follows: firstly, we present the reparameterization process and results of the new ReaxFF. Afterwards, we demonstrate how the enhanced force field improves the representation of the crystal structure of the LiF inorganic salt. We then proceed to test the ability of the enhanced force field to accurately describe the lithium mobility in LiF and highlight how it provides a more realistic diffusivity value for the Li atoms. Then we present a deep investigation of the results from the new ReaxFF, discussing the challenging aspects of this kind of potential. In the subsequent section, conclusion are drawn and a possible future outlook provided. Finally, the methods used are outlined in the final section.

**Table 1:** List of the most commonly used and useful Python tools and libraries in computational materials science.

	Description	PreP	Run	PostP
ASE^51,52^	Atomic Simulation Environment (ASE) is a Python library that provides a versatile framework for configuring, running, visualizing, and analyse atomistic simulations. Thanks to the object Calculator ASE provides a powerful interface to different codes.	$\checkmark$	$\checkmark$	$\checkmark$
Pymatgen^53,54^	Python Materials Genomics (Pymatgen) is a library for materials analysis, with a particular focus on solid-state studies and extensively used to produce and collect the data for the Materials Project (MP)^59^ database. It has functions for reading and manipulating structural, thermodynamic, and electronic properties of materials. It can also handle inputs and outputs from various DFT codes and easily access the MP crystallography database via its integrated API.	$\checkmark$		$\checkmark$
AiiDA^60,61^	Automated interactive infrastructure and Database (AiiDA) is an open-source, Python-based workflow management platform. Its main objective is to assist researchers in organizing, automating, managing, sharing, and tracking their simulations, thus enabling the reproducibility of complex workflows in computational materials science. Its plugging interface allows for the management of simulations performed with various codes, and it is designed to be used in conjunction with the Jupyter web interface, making the whole study interactive and easy to share.	$\checkmark$	$\checkmark$	$\checkmark$
MDAnalysis^62,63^	It is a versatile Python library mainly focused on allowing the manipulation and analysis of MD trajectories. With support for various trajectory and system configuration formats, it simplifies the process of translating data into n-dimensional NumPy^64^ arrays for further analysis.			$\checkmark$
Crystal Toolkit^65^	It is a library and web application framework for handling, manipulating, and analysing crystal structures. It is widely used in the MP database as an interactively visualizing web tool.			$\checkmark$
Quippy^66,67^	It is the Python high-level interface to QUantum mechanics and Interatomic Potentials (QUIP)^68^. QUIP is a set of software tools for performing MD simulations that can be used as a plugin for other programs such as LAMMPS, CP2K^69^, and ASE.	$\checkmark$	$\checkmark$	
PyLammps^70,71^	Large-scale Atomic/Molecular Massively Parallel Simulator (LAMMPS) is a powerful classical molecular dynamics simulation code. The python package, which can be installed after code compilation, allows to manage the LAMMPS simulations through the lammps module, a wrapper for the code’s C-library API.		$\checkmark$	
PLAMS^72,73^	The Python Library for Automating Molecular Simulation (PLAMS) is the Python interface for the commercial code Amsterdam Modeling Suite (AMS)^74^. Depending on the code used, it allows the automation of a wide range of simulations, including geometry optimization, vibrational spectroscopy, molecular dynamics, monte carlo (MC) simulations, and many other types of computational studies.		$\checkmark$	$\checkmark$
ParAMS^55,56^	Parameter optimization for Atomistic and Molecular Simulations (ParAMS) is a specialized Python library included in the AMS. Its main purpose is to facilitate the optimization workflow to search parameters for empirical energy functionals such as ReaxFF and density functional based tight binding (DFTB).	$\checkmark$		

These libraries can be used in one or more phases of material in silico study: PreP = pre-processing, Run = simulation running, and PostP = post-processing.

## Results and discussion

### ReaxFF reparameterization

**Figure 1:**
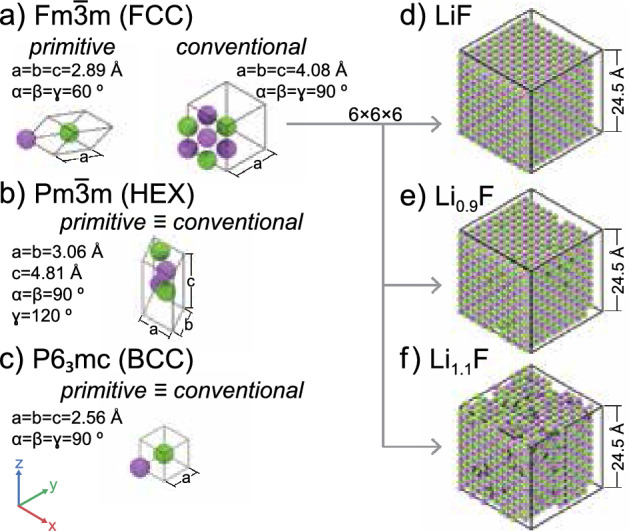
Isometric representation of unit cells used for ReaxFF reparameterization database generation and validation simulation boxes. The left column shows unit cells of LiF stable (**a**) and metastable phases (**b**,**c**) corresponding to face-centered cubic (FCC), hexagonal (HEX), and body-centered cubic (BCC) lattice types with space groups ${\text{Fm}}\bar{3}{\text{m}}$, ${\text{Pm}}\bar{3}{\text{m}}$, and ${\text{P6}}_3{\text{mc}}$ respectively^59^. The right column displays atomistic systems used for testing the improved force field built as supercells of the stable unit cell (**a**): equal Li-F atoms (**d**), 10% vacancies at Li sites (**e**), and 10% interstitial Li atoms (**f**). The shown renderings were generated using the Visual Molecular Dynamics (VMD) code^75^ and followed the Corey–Pauling–Koltun (CPK) colouring scheme^76^.

In the currently available ReaxFF force fields, all the energy contributions, including the Li-F interactions, were parameterized using a database of *ab initio* simulations specifically designed to capture the dissociation energy for potential reactions in the system. As a result, both the force field of Yun et al.^44^ and that of Wang et al.^46^ can predict the reaction products in Si-Li batteries with acceptable accuracy. However, the insufficient data on the crystal properties of inorganic salts restricts their ability to describe LiF aggregation and solid-phase transitions accurately. This limitation underscores the need for a new parameterization. In parametrizing the ReaxFF, the methodologies employed for data collection are essential. Tracing back to the foundational work by Van Duin et al.^39^, followed by the significant contributions of Chenoweth et al.^77^ in the context of hydrocarbons FF, the prevailing approach has been to leverage the functional forms of the reactive force field. This is typically achieved by utilizing DFT data from exploring diverse reaction pathways, varying environmental conditions, and frames captured from *ab initio* MD simulations. Such strategies ensure an accurate representation of molecular dissociation. However, prior studies have not specifically emphasized the quality assurance and correlation analysis of the collected data. Given our objective to integrate solid phase information into the ReaxFF, we developed a new database following the methodology proposed by LaBrosse et al.^78^. This methodology, initially tailored for developing force fields specifically for cobalt crystals, provides a robust framework for refining ReaxFF to precisely model solid-state phenomena. Furthermore, in this study, we have adopted a stochastic approach to select configurations. This strategy aims at minimizing autocorrelation and potential biases within the database, ensuring a more representative and accurate dataset for ReaxFF enhancement, and efficiently reduces the number of DFT calculations required for the database. A more exhaustive exploration, in contrast, might question the advantages of ReaxFF’s functional form even if it ensures a wider potential energy surface exploration. As a result, it is not always obvious if this method is more appropriate as compared to fully data-driven force field approach. Namely, the new database^79^ was created using stable and metastable crystalline unit cells from the Material Project database^59^ (Fig. 1). Several initial configurations were created using the Pymatgen and ASE libraries. Over 300 DFT simulations were performed using those systems, and all relevant quantities, such as energies, forces, and partial charges, were extracted for the objective function. In accordance with FAIR (Findable, Accessible, Interoperable, and Reusable) principles^80^, we stored all system and simulation data in an SQLite3 database using the ASE library. This database, along with metadata and clear usage instruction, has been shared on Zenodo (https://doi.org/10.5281/zenodo.7959121) and Github (https://github.com/paolodeangelis/Enhancing_ReaxFF_DFT_database) for easy accessibility and usability.

**Figure 2:**
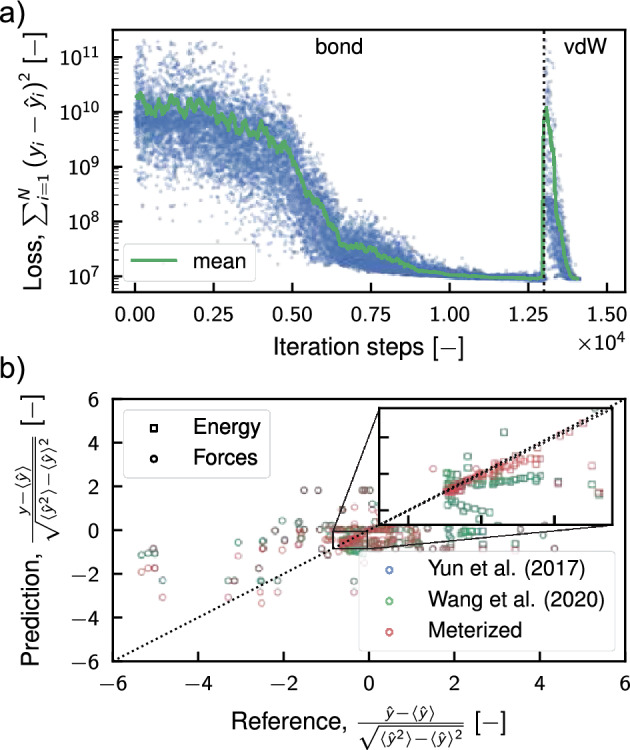
Evolution of the loss function during optimization of parameters and regression analysis. Panel (**a**) displays the loss function at each optimization iteration, represented by blue points. A green line indicates the moving average of the loss function, calculated using a 100-iteration window. The vertical dashed line marks the transition between optimizing different subsets of parameters, specifically the bond interactions (bond) and the van der Waals interactions (vdW). Panel (**b**) presents the regression results in a scatter plot format, contrasting reference values with predicted values for energy (square markers) and forces (circle markers). Predictions were computed using ReaxFF parameterizations by Yun et al.^44^, Wang et al.^44^, and our novel reparameterization (represented in blue, green, and red, respectively). Due to the distinct nature and wide range of these quantities, standardization was performed using the reference mean, $\left\langle \hat{y} \right\rangle$, and standard deviation, $\sqrt{\left\langle \hat{y}^2 \right\rangle - \left\langle \hat{y} \right\rangle ^2 }$, enabling legible visualization on the plot.

**Figure 3:**
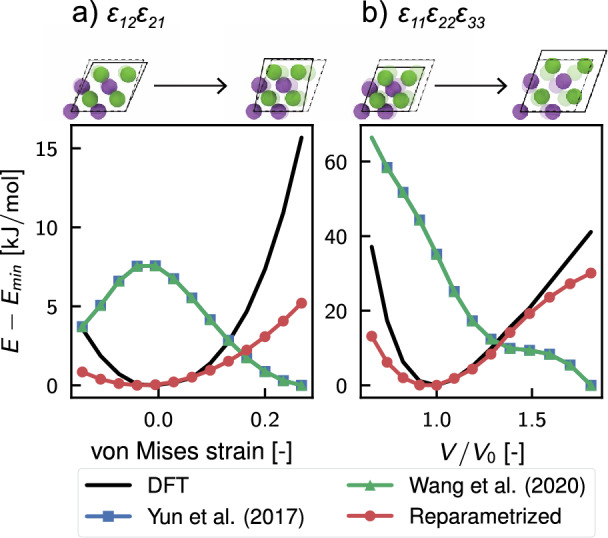
Exploring the stability of LiF under stress: A dual analysis reveals the material’s response to mechanical manipulation. In (**a**), the energy-strain plot displays the total energy changes with applied shear strain $\varepsilon _{12}=\varepsilon _{21}$, while (**b**) depicts the equation of state, i.e., the energy variations versus volume deformation ratio $V/V_0$ ($V_0$ is the volume at the equilibrium). The black line represents DFT results used for training, while the blue, green, and red lines illustrate the energy predictions made by the ReaxFF from Yun et al.^44^, Wang et al.^46^, and the proposed new reparameterization, respectively. The simulation snapshot above each plot illustrates the frontal view of the crystal at two extreme deformation cases, with the dashed black line indicating the minimal energy unit cell. Notice that green and blue lines are mostly overlapped in the reported pictures.

The ReaxFF coefficients were optimized using the Covariance Matrix Adaptation Evolution Strategy (CMA-ES)^81^ genetic algorithm. Generally speaking, this evolution algorithm searches for the optimal solution by sampling the population from a multivariate normal distribution (MVN). The generated samples are ranked based on the chosen loss function, and the best points, representing lower values for the loss function, are utilized to update the covariance and mean of the MVN. Then the updated MVN is used to obtain the next generation of samples, and the process continues iteratively until convergence or other predefined stopping criteria are met. In our case, the loss function is the sum of square errors (SSE) between values obtained from density functional theory calculations and molecular dynamics simulations. The algorithm converged after $1.3 \times 10^{4}$ iterations as shown in Fig. 2, thanks to the use of the step-wise optimization technique already employed by Wang et al.^82^. Indeed, similar to the study conducted by Wang et al.^82^, we capitalized on the physical-inspired structure of the ReaxFF force field, where parameters are categorized based on interaction and atom type, as outlined in Table 2. The approach consisted in gradually optimizing a selection of parameters relevant to particular types of interactions, instead than letting the algorithm operate in a very high-dimensional search space. Hence, we proceeded by optimizing initially the values associated with the bond interactions, then the values related to the dispersion energy, as evidenced by the leap in the loss function in Fig. 2a. However, after these first two subsets, further optimization of angle and torsional interactions was not feasible because all the initial guess values for their coefficients produced similar loss functions, which prevented the calculation of the second generation of parameters and covariance matrix for the CMA-ES algorithm. More information about the full protocol is provided in the methods section. The new parameterization has markedly enhanced the prediction accuracy for energy and forces within the database configurations, as evidenced by the regression plot in Fig. 2b, and the metrics detailed in Table 3 compared to the previously proposed ReaxFFs. This improvement has positively influenced the model’s capability to accurately describe the solid state of the inorganic salt, as demonstrated in Fig. 3. The figure includes an energy-strain curve (Fig. 3a) and Murnaghan equation of state (EOS) (Fig. 3b). The first plot was obtained by applying a shear strain $\varepsilon _{xy}$ to a $2\times 2 \times 2$ supercell of the stable LiF crystal. While the EOS was computed by expanding and contracting the system using a three-dimensional volumetric strain, since the two are connected by the following relation $\Delta V / V = \varepsilon _{xx} + \varepsilon _{yy} +\varepsilon _{zz}$. At a glance, it is clear that the previous parameterizations were unable to predict the condensed state of LiF accurately. Indeed, neither the force field developed by Yun et al.^44^, nor that of Wang et al.^46^ showed the presence of a minimum configuration in both the elastic strain and EOS cases. The shear deformation case was particularly problematic because the inverted concavity of the curves led to a nonphysical negative value for the elastic tensor component $c_{12}$, where the stress tensor is defined as $c_{ij}=\sigma _{ij}\varepsilon _{ij}^{-1}$ with $\varepsilon _{ij}$ representing the strain tensor and $\sigma _{ij}$ the stress tensor. The concavity of the energy-strain plot determines this component since it is the function of the second partial derivative of the energy with respect to strain, i.e., $c_{12} = V_0^{-1} \cdot \partial ^2 E /\partial \varepsilon _1 \partial \varepsilon _2$. On the other side, thanks to the newly designed database, the reoptimized ReaxFF force field accurately predicts equilibrium configurations for both EOS and elastic strain curves. Specifically, the prediction of the shear strain closely matches *ab initio* results in the region close to the equilibrium value, but deviates substantially for high shear strain values, indicating its limitations in predicting material stiffness accurately. Similarly, the ReaxFF prediction of the equation of state agrees well with the reference values, even for significant volume changes. This remarkable improvement of the proposed reparameterization over previous ones is also demonstrated for the one-dimensional deformation of the crystal and for the metastable crystals (as shown in supplementary Fig. S18). It is important to note, however, that the new force field predictions are less accurate for metastable cases with hexagonal and body-centered cubic lattice (Fig. 1), suggesting that the functionals used to describe various interactions may have limitations in generalizing the energies from the DFT simulations.

**Table 2:** Listing of ReaxFF coefficients for each section in the force field file, organized by interaction type.

Type	No. of parameters	Note
General	41	
Atoms	32	For each atom type $N_{A}$
Bonds	16	For each bond $N_{Bond} = \dfrac{(N_{A}+1)!}{(N_{A}-1)!2!}$
Off-diagonal	6	For each heterogeneous pair $N_{vdW} = \dfrac{N_{A}!}{(N_{A}-2)!2!}$
Angles	7	For each possible angle in the system $N_{Ang} \le \dfrac{(N_{A}+2)!}{(N_{A}-1)!3!}$
Dihedral	7	For each possible dihedral angle in the system $N_{Tors} \le \dfrac{(N_{A}+3)!}{(N_{A}-1)!4!}$
Hydrogen bonds	4	Usually only for O, C and N atoms

Please note that the numbers in the second column represent the number of coefficients per single entry (e.g., for a single atom type or a single bond). However, the total number of parameters required in the force field depends on the total number of atom types ($N_A$), as indicated in the third column.

**Table 3:** Comparative performance metrics for energy and force FF predictions on the training set.

FF	Energy		Forces	
	$\mathbf {R^2}$	RMSE $(\hbox {eV})$	${R^2}$	**RMSE** $\hbox {eV}$ Å$^{-1}$)
Yun et al.	$-0.093$	0.562	0.227	$5.1\times 10^{-3}$
Wang et al.	$-0.093$	0.562	0.227	$5.1\times 10^{-3}$
This work	0.293	0.452	0.377	$4.6\times 10^{-3}$

To evaluate the efficacy of the FFs we compute the Coefficient of Determination ($\textrm{R}^{2}$) and the Root Mean Square Error (RMSE) metrics.

### ReaxFF prediction of bulk LiF

**Figure 4:**
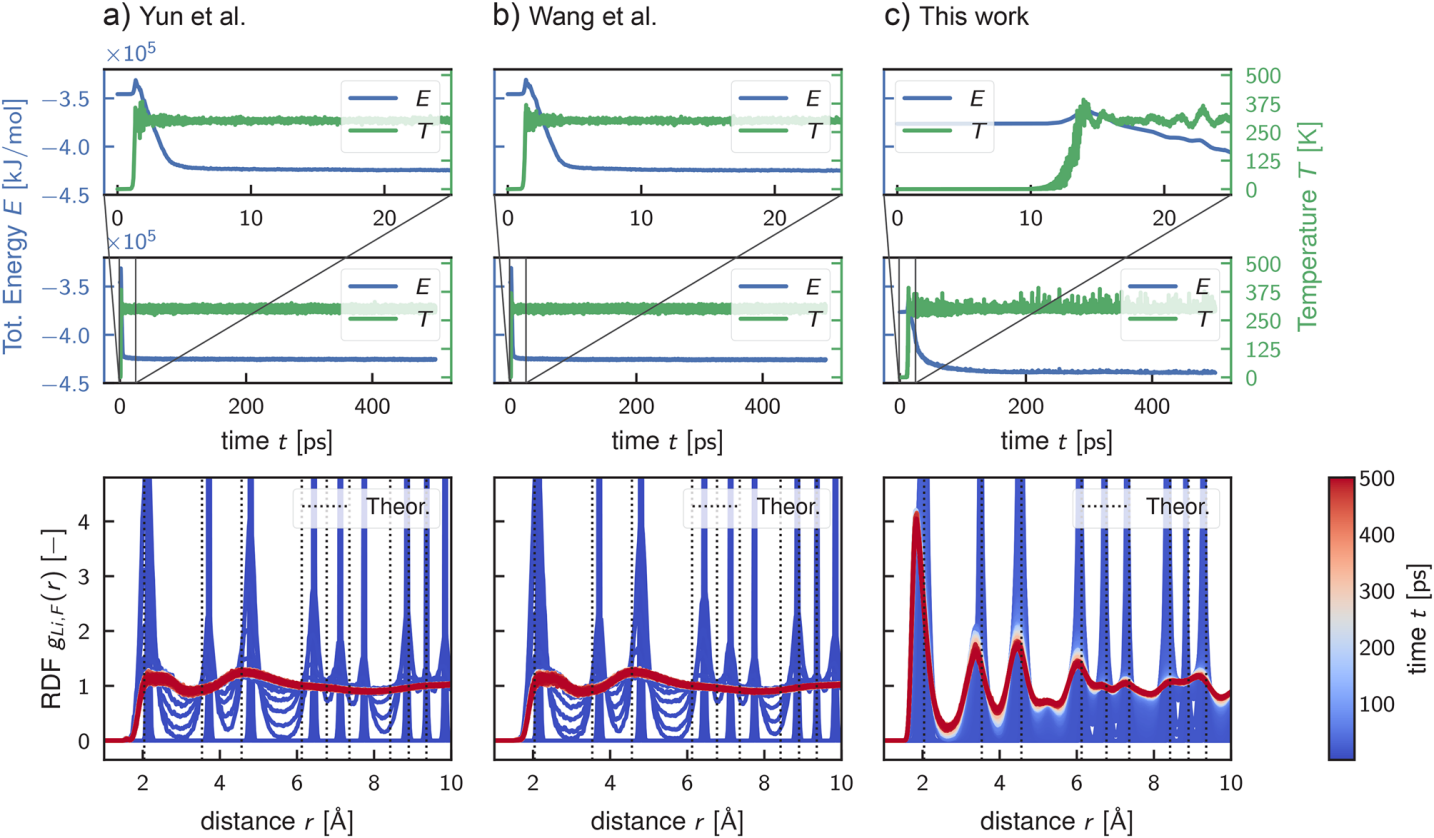
The evolution of bulk LiF simulations using reactive molecular dynamics at ${300}\,\hbox {K}$ using three ReaxFF: Yun et al.^44^ (**a**), Wang et al.^46^ (**b**), and the new reparameterization (**c**). The top graphs depict total energy trends (solid blue line) alongside temperature trends (solid green line), with inset plots providing detailed views of the early simulation trajectories. Conversely, the bottom panels show the time evolution of the Li-F radial distribution function (RDF). Each RDF curve is obtained by averaging the trajectory every ${0.1}\,\hbox {ps}$ and then coloured according to the simulation time indicated by the colour bar on the right. Notably, RDF curves from the literature force field resemble more systems in a liquid phase rather than a solid phase.

To validate the new parameterization, as first test case, we conducted reactive MD simulations using all the discussed ReaxFFs for systems with Li and F atoms to simulate bulk LiF at ambient conditions ($300\,\hbox {K}$, ${1}\,\hbox {bar}$). The simulation was performed on a relatively big system with respect to the ones used for the training. Indeed, a supercell of the LiF crystal created by replicating the conventional unit cell five times in all directions was placed in a simulation box of size $20.4 \times 20.4 \times 20.4$ Å$^{3}$, which is analogous to the system in Fig. 1d. After the initial relaxation of the system, including box size adjustment, the pure LiF crystal was simulated for ${500}\,\hbox {ps}$ under an NVT ensemble (constant number of atoms, volume, and temperature) set at ${300}\,\hbox {K}$ and with a time-step of ${0.25}\,\hbox {fs}$, and to attenuate possible artefacts due to the thermostat we have adjusted the relaxation time to be 1000 times the time-step (${250} \, \hbox {fs}$)﻿. In Fig. 4 we report the total energy, temperature, and radial distribution function (RDF) between the Li and F atoms, $g_{Li,F}(r)$, evaluated during the reactive MD simulations with different potentials. Comparing the results from the simulations using the Yun et al.^44^ and Wang et al.^46^ force fields (Fig. 4a and b), we observed similar behavior, indicating minimal differences in the parameters for Li and F used in both ReaxFF. Furthermore, we can observe that both force fields failed to accurately describe the simulated system, as the thermal agitation overpowered the binding energy, resulting in the amorphization of the inorganic salt when it reached the external heat bath temperature. This resulted in a sudden drop in the total energy at ${2}\,\hbox {ps}$ that results into an artificial phase change. Indeed, since the melting temperature of LiF is ${1121.35}\,\hbox {K}$ (${848.2}\,^{\circ }\hbox {C}$)^83^, the system should ideally exist in a solid phase in the simulated virtual conditions. This remarkable and unexpected result is even more evident looking at the two radial distribution functions in Fig. 4a and b, where the initial peaks due to the ordered structure of the system and immobility of the atoms disappear, and the curves become liquid-like RDF as the temperature rises. Moreover, we can notice a shift in the initial RDF peaks with respect to the theoretical position represented by the vertical dotted line, indicating an anomaly in the LiF solid state description of the two FFs. In contrast, our new parameterization performed well in simulating a simple bulk LiF system. Indeed, Fig. 4c shows that the radial distribution function has initial peaks that align with theoretical values and remain consistent even at high temperatures. In this case, the thermal agitation of the atoms resulted only in the smoothening of the peaks and the emergence of secondary peaks due to the oscillation of some atoms between the lattice and interstitial positions. This important improvement is also evident by visualizing the temperature growth, which gradually reaches ${300}\,\hbox {K}$ and is held constant as the system converges to an equilibrium state, as visible from the total energy (Fig. 4c).

### Diffusion of Li in LiF prediction from ReaxFF

**Figure 5:**
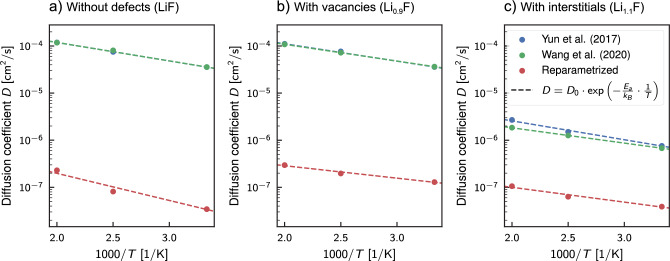
Arrhenius plot displaying the diffusion coefficient of Li (*D*) in pure bulk LiF ($\textrm{LiF}$) and with point defects of vacancy ($\textrm{Li}_{0.9}\textrm{F}$) and interstitial ($\textrm{Li}_{1.1}\textrm{F}$) types, obtained through the molecular dynamics simulations. The blue, green, and red dots represent the *D* values obtained using the Yun et al.^44^, Wang et al.^46^, and the proposed new ReaxFF parameterization, respectively. The drawn dashed line results from a linear regression of the coefficients using the least square method.

In designing the training set, we aimed to enhance the accuracy of predicting both the mechanical and thermophysical properties of the inorganic material through ReaxFF simulation. To achieve this, we included configurations with point defects like vacancies, interstitials, and substitutions and incorporated data from *ab initio* simulations conducted at different temperatures. Therefore, the second test case for the new ReaxFF was to compute the lithium transport properties with a campaign of MD simulations. We evaluated the mass diffusivity of Li atoms in three systems illustrated on the right side of Fig. 1, specifically: one with ${10}\,\%$ interstitial lithium atoms ($\textrm{Li}_{1.1}\textrm{F}$), one with ${10}\,\%$ lithium vacancies ($\textrm{Li}_{0.9}\textrm{F}$), and one with no defects ($\textrm{LiF}$). These systems were simulated for ${500}\,\hbox {ps}$ at three different NVT temperatures (${300}\,\hbox {K}$, ${400}\,\hbox {K}$, and ${500}\,\hbox {K}$). The lithium diffusion coefficients were extracted by measuring the mean square displacement (MSD) during the simulation and by using the Einstein-Smoluchowski diffusion equation^84–86^:

1 $$\begin{aligned} \lim _{t \rightarrow \infty } \langle \Vert \textbf{r}_i(t) - \textbf{r}_i(0)\Vert ^2 \rangle _{i \in Li} = c_{d} D t, \end{aligned}$$

where the limit argument is the MSD definition, *t* is time, *D* is the diffusion coefficient, and $c_{d}$ is a constant indicating dimensionality, with values of 2 for one-dimensional, 4 for two-dimensional, and 6 for three-dimensional diffusion. We obtained the diffusion coefficient values by determining the slope of the straight line that best fits the MSD evolution for each simulation (as seen in the supplementary Figs.  S19–S21). In Fig. 5, we plotted them on a logarithmic scale and used the inverse of the temperature as abscissa. This enabled us to extract the activation energy that appears in the Arrhenius equation:

2 $$\begin{aligned} D = D_0 \cdot \exp \left( - \dfrac{E_a}{k_B T} \right) . \end{aligned}$$

Here, *D* is the lithium diffusion coefficient from the MD simulations, $D_0$ is the maximum diffusivity value (the limit value at infinite temperatures), $k_B$ is the Boltzmann constant, and $E_a$ is the activation energy. We repeated this process for all three ReaxFFs discussed, and the resulting values of $D_0$ and $E_a$ are reported in Table 4. The new force field provides an improved description of bonding forces, which results in a significant reduction in the mobility of lithium atoms within the crystal lattice, as expected. For example, in the case study of defect-free LiF supercell at ${300}\,\hbox {K}$, the diffusion coefficient predicted by both the Yun et al.^44^ and Wang et al.^46^ force fields is $3.56 \times 10^{-5}\,\hbox {cm}^{2}/\hbox {s}$, which is significantly closer to the diffusivity of lithium ions in the electrolyte ($0.5\times 10^{-5}- 1.4 \times 10^{-5}\,\,\hbox {cm}^{2}/\hbox {s}$^87^) rather than in the solid. In contrast, the new parameterization predicts a diffusivity of $3.44 \times 10^{-8}\,\hbox {cm}^{2}/\hbox {s}$, which is a reduction of 3 orders of magnitude. Similar reductions in diffusivity are seen in cases involving interstitials and vacancies.

**Table 4:** The values of the activation energy, $E_a$, and the maximum value of the lithium self-diffusion coefficient, $D_0$, for the three simulated systems and for each ReaxFF used. The errors were estimated based on the inference for the linear regression model coefficients (least squares method), and assuming a ${95}\,\%$ confidence interval.

	**ReaxFF**	$\textbf{D}_\textbf{0}$ $[\hbox {cm}^{2}/\hbox {s}]$	$\textbf{E}_\textbf{a}$ $[\hbox {kJ}\,\hbox {mol}^{-1}]$
$\textrm{LiF}$	Yun et al.	$(7.23 \pm 0.05) \times 10^{-4}$	$7.50 \pm 0.02$
	Wang et al.	$(7.2 \pm 0.5) \times 10^{-4}$	$7.0 \pm 0.2$
	This work	$(3 \pm 2) \times 10^{-6}$	$11.0 \pm 1.6$
$\textrm{Li}_{0.9}\textrm{F}$	Yun et al.	$(6.1 \pm 0.3) \times 10^{-4}$	$7.1 \pm 1.4$
	Wang et al.	$(5.5 \pm 0.2) \times 10^{-4}$	$6.810 \pm 0.013$
	This work	$(9.0 \pm 1.7) \times 10^{-7}$	$5.1 \pm 0.5$
$\textrm{Li}_{1.1}\textrm{F}$	Yun et al.	$(1.5 \pm 0.3) \times 10^{-5}$	$7.6 \pm 0.5$
	Wang et al.	$(8.2 \pm 1.3) \times 10^{-6}$	$6.22 \pm 0.04$
	This work	$(4.0 \pm 1.2) \times 10^{-7}$	$6.0 \pm 0.8$

**Figure 6:**
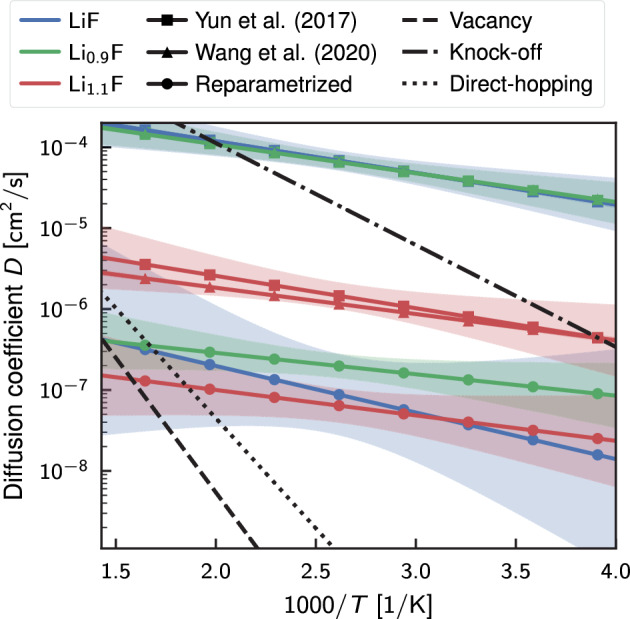
Arrhenius plot of diffusion comparing the predictions from Reactive MD and DFT simulations. The interpolation from MD results is shown using continuous lines and markers (square for Yun et al.^44^, triangular for Wang et al.^46^, and circular for the new reparameterization), and the confidence interval of 95% is shown with the shaded areas. The effect of defects on diffusion is indicated by different colors: blue color for the defects-free case, green for the cases with vacations, and red for the case with interstitial lithium. The black dashed, dash-dot, and dotted lines represent the Arrhenius curves estimated by Zheng et al.^31^ for three potential transport mechanisms (vacancy, knock-off, and direct-hopping) from Climbing Image Nudged Elastic Band (CI-NEB) studies.

We compared the obtained values with *ab initio* values calculated by Zheng et al.^31^. They studied lithium diffusion in various organic components of the SEI by exploring the energy surface using the surface energy Climbing Image Nudged Elastic Band (CI-NEB) method^88^. The exploration of the energy surface by Zheng et al. confirmed that the diffusion of lithium in lithium fluoride occurs via three distinct possible mechanisms. These mechanisms include: vacancy movement, where lithium atoms jump to the adjacent empty lattice site (vacancy); direct-hopping, where lithium moves directly from one interstitial site to another; and the knock-off mechanism, in which an interstitial lithium atom replaces a lithium atom in the crystal lattice, thus causing the displaced lithium atom to move into another interstitial site. Figure 6 shows the Arrhenius curves for the three diffusion mechanisms calculated using the values of activation energy and maximum diffusion obtained from the *ab initio* study, as well as the curves obtained from the reactive molecular dynamics simulations. Regarding the maximum diffusion coefficient $D_0 = 3\alpha ^2 v$, Zheng et al. approximate its value using the estimated phonon frequency in the crystal $v=1 \times 10^{13}\,\hbox {s}^{-1}$ and the migration distance for the lithium ions $\alpha$ observed during the CI-NEB simulations^31^. Molecular dynamics simulations can exhibit various diffusion mechanisms, leading to the calculated curves for the new force field and each studied system falling within the region defined by the three diffusion mechanisms. For the calculated temperature values, the diffusion coefficients turn out to be intermediate values between pure knock-off diffusion and pure direct-hopping diffusion. However, looking at the slope of the Arrhenius curves predicted by molecular dynamics simulations in Fig. 6 it is clear that even the new ReaxFF underestimated the activation energy. Indeed, even for the most favorable diffusion mechanism (knock-off), the activation energy obtained from the *ab initio* energy profile is ${24.1}\,\hbox {kJ}\,\hbox {mol}^{-1}$, which is more than double the value calculated with the new force field for the defect-free case (${11.0}\,\hbox {kJ}\,\hbox {mol}^{-1}$). The discrepancies are even more significant for all other cases listed in Table 4.

**Figure 7:**
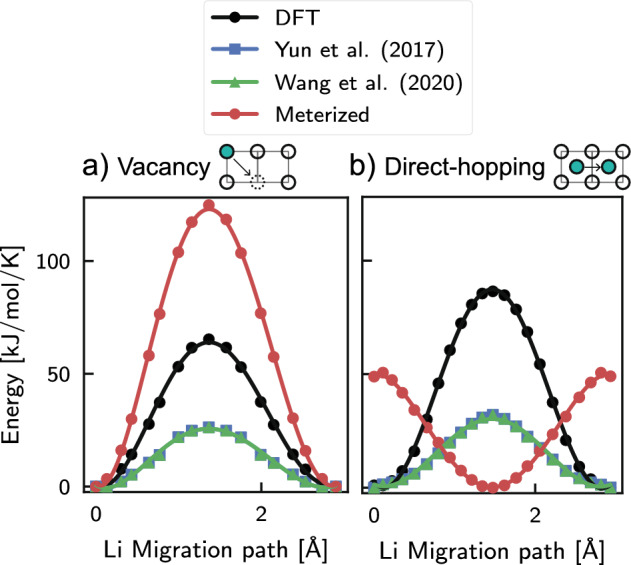
Graphical visualization of the energy profile for lithium migration by vacancy (**a**) and direct-hopping (**b**) calculated by CI-NEB is depicted by the black line and circular markers. The markers indicate the exact energy value of each distinct image obtained from the convergence of the CI-NEB algorithm and were used to calculate the energy using three different methods: ReaxFFF by Yun et al.^44^, Wang et al.^46^, and a new reparameterization, represented by blue, green, and red colored markers and line, respectively.

To address this discrepancy, we conducted further analysis of the ReaxFF by reproducing the energy curves for lithium diffusion by vacancies and by direct-hopping using the CI-NEB method. Initial and final configurations were made using a $2\times 2 \times 2$ LiF supercell and inserting the lithium atoms into two adjacent sites identified with the Voronoi analysis from the pymatgen python library^53^. While for the vacancy diffusion case, one lithium was removed from the crystal lattice from two adjacent unit cells. The energy profile was then obtained using the CI-NEB algorithm with the same parameters used in the DFT simulation for training the new ReaxFF. To ensure convergence, we used 23 images for direct-hopping diffusion and 17 images for vacancy diffusion. Our simulations yielded activation energies of ${64.3}\,\hbox {kJ}\,\hbox {mol}^{-1}$ for vacancy diffusion and ${87.5}\,\hbox {kJ}\,\hbox {mol}^{-1}$ for direct-hopping. The value for vacancy diffusion was comparable to previous work (${63.7}\,\hbox {kJ}\,\hbox {mol}^{-1}$). Still, the value for direct-hopping is higher than that calculated by Zheng et al. (${52.1}\,\hbox {kJ}\,\hbox {mol}^{-1}$) due to the use of a smaller system to reduce the computational cost, resulting in a higher defect density in our case. Subsequently, from the result obtained from the DFT simulations, we proceeded to calculate each image’s energy using the various ReaxFF discussed. In Fig. 7 we show the comparison of the energy profile predicted by the various methods.

The additional investigation of ReaxFF presented in Fig. 7 reveals significant discrepancies between previous parameterizations, DFT prediction, and the reoptimized force field. Specifically, the energy barrier value prediction was found to be incorrect for all the ReaxFF studied, and the new force field exhibited unrealistic behavior in the case of interstitial diffusion. Indeed, the maximum energy values corresponded to the initial and final configurations, while the minimum value corresponded to the transition configuration. This behavior contradicts the physical nature of the phenomena, and the predictions made by the DFT calculation and even by previous parameterizations by Yun et al.^44^ and Wang et al.^46^. In order to be able to explain this numerical artifact, it is important to consider the general structure of ReaxFF, which is described by the provided equation:

3 $$\begin{aligned} \begin{aligned} V_{reaxFF}&= \underbrace{ V_{bond} + V_{angle} + V_{dihedral} }_{\text {bonded interaction}} \\&\quad + \underbrace{ V_{over} + V_{under} }_{\text {bonded interaction (coordination)}} + \underbrace{ V_{vdW} + V_{q} }_{\text {non-bonded interaction}} \\&\quad + \underbrace{ V_{conj} + V_{trip} + V_{C_2} + V_{H-bond} + V_{lp} }_{\text {system specific}}. \end{aligned} \end{aligned}$$

$V_{reaxFF}$ represents the total reactive potential of the particle *i* interacting with neighboring atoms, which can be divided into three contributions: bonded, non-bonded, and “system specific”. The first describes the bonded interaction between atoms and is formed by the bond energy $V_{bond}$, the angle energy $V_{angle}$ and the dihedral (or torsional) energy $V_{dihedral}$ and the corrective terms over-coordination $V_{over}$, and under-coordination $V_{under}$ energies. The non-bonded energy, which is analogous to classical molecular dynamics, is given by the sum of the van der Waals potential $V_{vdW}$ and Coulomb potential $V_{q}$. The last terms, on the other hand, are a set of corrective energies that accounts for specific phenomena in certain specific systems. For additional details about the mathematical and physical implications of each term, the reader can refer to the works by Van Duin et al.^39^ and Chenoweth et al.^77^.

From Eq. (3) and the previous studies, we can attribute the unrealistic behavior observed with the new force field to the increased energy contribution from bond energy, which improves the description of system connectivity. However, this results in an increase in the effect of correction energy terms, specifically over-coordination energy $V_{over}$, and under-coordination energy, $V_{under}$, in Eq. (3), which are functions of the difference between the total number of bonds of an atom and its valence number^39^. Indeed, in the initial and final conditions, the lithium atom has the same number of neighboring atoms, which is higher than in the transition state, where space is created by moving the lattice atoms, and the increase of the lithium distance respects the atoms at the extremes of the unit cell. This unexpected artifact highlights the critical importance of carefully considering energy contributions in force fields to ensure accurate predictions, as observed in the case study using ReaxFF. Consequently, it is likely that additional configurations are needed to be included in the training set. However, it is important to note that this alone does not guarantee the resolution of all the aforementioned issues. In fact, due to the reliance on a fixed functional shape in the force field, a more deep and sophisticated alteration in the mathematical expression of the energy terms might be required. Implementing such changes would involve modifying the code, which exceeds the scope of this work. This also highlights that a critical challenge here is the limited transferability of the parameterization beyond the specific database, emphasizing the necessity of a comprehensive database of *ab initio* simulations that is specifically tailored and adequately representative for studying the targeted system at an atomistic scale.

## Conclusion

In conclusion, the proposed partial reparameterization methodology for the ReaxFF has significantly improved the description of lithium fluoride in the solid state, leading to better predictions of its solid phase properties and lithium mobility in LiF crystal using reactive MD simulations. Indeed, implementing the new parameters of the ReaxFF has demonstrated its ability to predict the stable unit cell under mechanical deformation accurately, exhibit typical solid-state RDF, and notably reduce lithium diffusivity in LiF by at least two orders of magnitude. The automation and interactivity of the protocol, achieved by leveraging Python libraries for atomistic simulations, made it possible to construct and simulate various configurations of the LiF crystal needed to build a database for correcting bond and van der Waals interactions of the ReaxFF. The new force field obtained from the re-optimization not only improved the behavior of the crystal in the solid phase but also partially corrected the description of lithium transport phenomena in LiF.

However, the in-depth investigation carried out revealed that the diffusion activation energies predicted by the new force field are still underestimated. This limitation may be due to the method used to construct the database, which did not directly sample lithium transport, but focused more on local or global deformation of the crystal lattice. This study highlights the strong dependence of the ReaxFF on the configurations included in the database. Hence, future effort should focus in addressing the challenges associated to the effective interpolation of the energy surface in unexplored or underrepresented regions in the adopted training set. The need to tailor ReaxFF for each specific scenario presents a significant challenge, especially when considering the upscaling of electronic simulations, such as DFT, to the molecular level. A common mitigation strategy involves utilizing previous parametrizations to define the boundaries for parameter optimization. However, for less commonly explored elements, like Li and F in our case, other parametrizations might be nearly identical and not particularly useful. Therefore, in the absence of disparate parametrizations in the literature to effectively constrain the search space, a convenient approach might be to rely on an extensive database. Nevertheless, based on our experience, as far as the explored case study is concerned, the need for ever-larger databases poses serious questions on the cost-benefit ratio of this method. Although the ReaxFF MD simulations are much faster than *ab initio* simulations, given all the functionals to be computed and the need to update the charges at each step reduces by at least a factor of 100 the speed of reactive MD simulations as compared to simulations with Lennard-Jones fluid^89^.

In addition to the sensitivity of the ReaxFF to the training set used, the difficulty in accurately describing the system studied in this work may also be attributed to an intrinsic bias of the force field. In particular, it is worth noting that the original formulation of the ReaxFF potentials proposed by Van Duin in 2001 was validated primarily on organic systems^39^, and various new functionals have been added to improve the accuracy and applicability of ReaxFF in other systems over the past few decades^90–92^. These include the “lone-pair” energy term ($V_{lp}$ in Eq. (3)) for hydrocarbon combustion^77,90,93^, a three-body functional ($V_{trip}$ in Eq. (3)) term for $\mathrm {-NO_2}$ group chemistry^90,94,95^, and an energy term ($V_{H-bond}$ in Eq. (3)) to account for hydrogen bonds in aqueous systems^77,96^, among others. Therefore, it may be necessary to introduce new correction energy terms to improve the accuracy of ReaxFF in strongly inorganic systems such as LiF.

On the other hand, the rapid development of machine learning-based (MLFF) and neural network-based force fields^97–101^ (NNFF) may provide alternative and accurate approaches to the ReaxFF. The database constructed using the proposed protocol (or even further automatized by algorithmic orchestration^102^) could be used for training these new force fields, which may provide superior performance, possibly at the cost of compromising on the physical insight into the parameters obtained from the training. With their flexibility and high interpolation capabilities, these newly developed force fields^101,103,104^ offer promising solutions to the challenges identified in this work and with the ReaxFF. These force fields describe the chemical local environment using a feature transformations (“descriptors”) algorithm^99,105,106^ instead of the bond order and functional parameters utilized in ReaxFF. However, they confront a limitation common in machine learning applications for materials science, i.e. the high dimensionality of features relative to the small sample size. This issue has been thoroughly reviewed by Liu et al.^107^, who also suggest approaches like feature reduction and sample augmentation to enhance model performance and accuracy. In summary, the proposed methodology, coupled with the synergistic approach to data quantity governance^107^, could be extended to the parameterization of various other potentials, and by increasing the number of initial configurations, it may also be possible to proceed with the parameterization of neural network potentials. Moreover, the guided reparametrization method could incorporate other frequently encountered inorganic compounds in the SEI within future ReaxFF or NNFF. This expansion offers an exciting opportunity to explore increasingly intricate and realistic systems resembling mosaic structures^108^ including also other SEI compounds (e.g. $\textrm{Li}_{2}\textrm{CO}_{3}$, $\textrm{Li}_2\textrm{O}$) thus emulating the Peled model^7^.

## Methods

### Interactive reparameterization protocol

**Figure 8:**
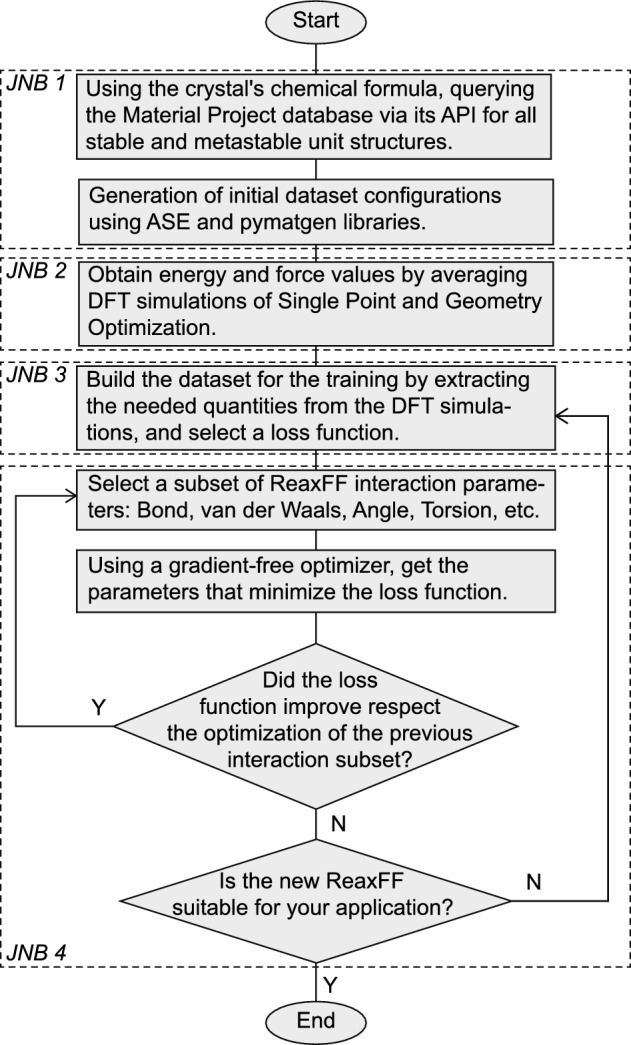
Flowchart followed for the reparameterization of ReaxFF. The dotted boxes indicate the Jupyter Notebook (JNB) specific to that part of the workflow, all available at https://doi.org/10.5281/zenodo.8036775 and in the repository https://github.com/paolodeangelis/Enhancing_ReaxFF.

The protocol for calculating the new ReaxFF parameters is presented in a flowchart shown in Fig. 8. The procedure is carried out using four Jupyter Notebooks (JNBs)^57,58^ that facilitate the automatic construction, visualization, and simulation of the necessary configurations for database construction and optimization of the reactive potential. Python libraries ASE^51,52^, pymatgen^53,54^, PLAMS^72,73^ and ParAMS^55,56^, designed for atomistic systems manipulation and simulation are utilized throughout the entire process, allowing for streamlined and efficient handling of the various steps.

The first JNB initiates the protocol by defining the chemical formula of the component to study. Then, using the API from the Materials Project database, the available crystalline units are downloaded. In the case of LiF, there are three configurations available, corresponding to different lattices (Fig. 1a–c), one stable (${\textrm{Fm}}{\bar{3}}{\textrm{m}}$) and two metastable ($\textrm{P6}_{3}\textrm{mc}$, ${\textrm{Pm}}{\bar{3}}{\textrm{m}}$). These crystals are imported into the interactive work environment as virtual objects from the ASE (Atoms) and pymatgen (Structure) libraries. Within this virtual environment, the crystals were manipulated to generate initial configurations for the DFT simulations. Specifically, we build different defected or deformed crystals following the procedure by LaBrosse et al.^78^, namely:

**Supercells**: To capture the effect of the atoms’ coordination in the training set, several supercells of size $2\times 1 \times 1$, $2\times 2\times 1$, $2\times 2 \times 2$, $3\times 2 \times 2$, $3\times 3 \times 2$, and $3\times 3 \times 3$ were constructed.
**Vacancies**: We randomly removed a lithium or fluorine atom from the $3\times 2 \times 2$ supercell, up to a maximum of five vacancies.
**Strain**: We applied different types of strain to each LiF crystal, including a normal strain $\varepsilon _{11}$, a shear strain $\varepsilon _{12}=\varepsilon _{21}$, and a homogeneous deformation in all directions $\varepsilon _{11}=\varepsilon _{22}=\varepsilon _{33}$ (needed for computing the equation of state), resulting in 13 configurations with strain values ranging from $- 12.5 \%$ to $23.5 \%$.
**Substitution**: We randomly substituted a lithium or fluorine atom with the opposite species, with a maximum of five possible defects for each crystal.
**Interstitial**: We inserted an interstitial atom at the center of Voronoi volumes obtained from the $3\times 2 \times 2$ supercell for each LiF crystal. We repeated this procedure five times to create structures with 1, 2, ..., and 5 interstitial atoms.
**Slab**: To include surface energy in the ReaxFF, we generated crystal slabs for the surfaces (100), (110), (111), and (210). We repeated each slab 2, 3, or 4 times to account for different thicknesses and we introduced sufficient empty space along the normal direction to avoid numerical artifacts due to the periodic boundary conditions (PBCs).
**Bulk at 300 K and 500 K**: We included frames of *ab initio* MD simulations at ${300}\,\hbox {K}$ and ${500}\,\hbox {K}$ for the $3\times 2 \times 2$ supercell to predict possible energy fluctuations during ReaxFF MD.
**Amorphous LiF**: To account for the high-energy state of the crystal, we included possible amorphous LiF with an *ab initio* MD simulation at $T={2500}\,\hbox {K}$.

In the second JNB, previously constructed configurations are used to perform *ab initio* simulations to obtain the numerical values needed for the databases. The ASE and pymatgen objects of the various systems were used as input to the PLAMS library of the AMS^74^ commercial code, enabling the running, control, and processing of DFT simulations. For LiF, over 300 DFT simulations were carried out using the BAND plane-wave DFT code available in AMS, as listed in the supplementary Table S1. Single-point (SP) simulations were used to calculate the energy and force values of each atom in each configuration, by solving the Kohn-Sham equations while considering the atom cores as fixed. For systems with defects, geometry optimization (GO) simulations were initially performed to determine the equilibrium configuration. *Ab initio* MD simulations were performed using Grimme’s extended density functional based on tight-binding (DFTB)^109^ due to their low numerical cost. From the resulting trajectories, 10 frames were selected, and their force and energy values were refined using SP-DFT simulations.

The third and fourth JNBs heavily rely on the AMS ParAMS^55,56^ library, which is specifically designed for potential optimization. In the third JNB, all quantities, such as energy, forces, and charges needed for the database, are extracted, resulting in more than 3000 entries. Regarding the potential energy, because it is a state function, the database does not include the absolute values obtained from the DFT simulations but the formation energy approximated by the relative energy obtained with respect to the defect-free configuration of the LiF crystal, namely:

4 $$\begin{aligned} \Delta H_i \approx \Delta E_i = E\left( X_{i}^{LiF}\right) - \left[ E\left( X_{bulk}^{LiF}\right) + n_i \cdot \mu _{Li} + m_i \cdot \mu _{F}\right] . \end{aligned}$$

Where $\Delta H_i$ is the formation energy of *i*-th configuration, $E\left( X_{i}^{LiF}\right)$ represents the energy of the *i*-th configuration, $E\left( X_{bulk}^{LiF}\right)$ is the energy of the bulk LiF configuration, $n_i$ and $m_i$ are integer numbers of Li and F atoms in the *i*-th comparared to the bulk and their sign depend on whether we removed or added atoms, while $\mu _{Li}$ and $\mu _{F}$ chemical potential, approximated by the energy per atoms obtained simulating pure Li and F unit cells.

This database is then used to optimize the ReaxFF in the fourth and final JNB. The optimization process begins by selecting the group of parameters that will form the search space, starting with those that significantly influence the behavior of the ReaxFF, such as the bond energy, followed by the van der Waals energy and angular energy terms. Various gradient-free optimization algorithms are available in the ParAMS library, and for the ReaxFF optimization for LiF, we chose the genetic algorithm Covariance Matrix Adaptation Evolution Strategy algorithm (CMA-ES)^81^ to minimize the objective function represented by the sum of squared errors (SSE) between the DFT values and the ReaxFF values.

Each group or subset of parameters is optimized sequentially, and the ReaxFF optimization process is considered complete when further improvements in the loss function become negligible.

### Density functional theory calculations

The dataset values (energies, forces, charges, etc.) for the ReaxFF optimization were obtained from DFT simulations performed with the commercial BAND^110,111^ code available in the AMS suite. We numerically solved the Kohn-Sham equations using the Perdew-Burke-Ernzerhof (PBE)^112^ functional and the polarized double zeta (DZ) numerical atomic orbitals (NAOs) basis set for the calculation of the *s*, *p*, and *d* orbitals. The software automatically chose the values of k-point and frozen electrons depending on the desired accuracy, and we selected a high accuracy value that guaranteed an error of less than ${0.01}\,\hbox {eV}$ per atom, and by comparing the formation energy values obtained for each crystalline unit of LiF studied with the values reported on the Material Project online database (see Fig. 1a–c). For very inhomogeneous systems, such as those with a large number of interstitial atoms or surfaces, we calculated forces without frozen atoms and using a single zeta (SZ) type basis set to speed up the calculation of the equilibrium configuration. We then refined the resulting configuration using the settings described above.

For detailed instructions on installing and utilizing the protocol and database repository, please refer to the supplementary material provided.

### Molecular Dynamics calculations

All the reactive molecular dynamics simulations were performed using the open-access code LAMMPS^70^ with the ReaxFF package^113^. The initial configuration for the diffusion of Li in bulk LiF was obtained by starting from the primitive unit cell of the stable crystal obtained from the Material Project database. The unit cell was then converted into the conventional unit cell using the pymatgen routine ConventionalCellTransformation^53^ and replicated six times along all directions to obtain a $6 \times 6 \times 6$ supercell with final dimensions of 24.5 Å$\times {24.5}$ Å $\times {24.5}$ Å. To create the system with vacancies (i.e. $\textrm{Li}_{0.9}\textrm{F}$), 86 randomly selected lithium atoms were removed. While to create the system with interstitial atoms (i.e. $\textrm{Li}_{1.1}\textrm{F}$), 86 lithium atoms were placed inside the LiF supercell using the PACKMOL code^114^. After the initial energy minimization, the system was simulated for ${0.5}\,\hbox {ns}$ using an NVT ensemble at three different temperatures (${300}\,\hbox {K}$, ${400}\,\hbox {K}$, and ${500}\,\hbox {K}$) with a Nose-Hoover thermostat^115^ and a relaxation time of $\tau _T = {25}\,\hbox {fs}$. The integration time step was set to $\delta t= {0.25}\,\hbox {fs}$. Thermodynamic properties, including the instantaneous mean square displacement for all lithium atoms, were sampled every 50 simulation steps to compute the diffusivity as discussed later.

### Diffusion energy barrier calculations (DFT)

To determine the energy barrier of Li ion diffusion, we used the climbing image-nudged elastic band (CI-NEB) method^88^. To employ this method, we built a $2 \times 2 \times 2$ supercell of the primitive cell of the stable LiF crystal and placed a Li atom in the interstitial site found with the Voronoi analysis^116^ in two adjacent cells to study the diffusion by interstitials. While, for the vacancy case, we removed a Li atom from two adjacent cells of the supercell. After geometry optimization of the initial and final states, we performed the CI-NEB calculation using 23 images for the direct-hopping diffusion case and 17 images for the vacancy case. We set the maximum perpendicular force component for the transitional state to be $2.5\,\hbox {eV}$ Å$^{-1}$ as the climbing convergence criterion. Finally, we obtained the activation energy by averaging the energy differences between the initial and transitional state and the final and transitional state.

### Diffusion energy barrier calculations (MD)

To compute the diffusion coefficient *D* for each combination of temperature, system, and potential, we employed the Einstein-Smoluchowski diffusion equation^84–86^, Eq. (1), that requires the time derivative of the MSD obtained from ReaxFF MD simulations. We obtained the numerical MSD from the MD trajectories and fitted it to a linear model of the form $\langle \textbf{r}^2 \rangle = \alpha _0 + \alpha _1\cdot t + \varepsilon$, where $\langle \textbf{r}^2 \rangle$ represents the mean-square displacement, $\varepsilon$ is the statistical error, and *t* denotes the elapsed simulation time (Figs. S19–S21). To determine the coefficients $\alpha _0$ and $\alpha _1$, we used the least squares method (LSM)^117^. Consequently, the diffusivity can be evaluated as $D = \alpha _1 / 6$.

An analogous procedure was followed to compute the activation energy from the Arrhenius law, Eq. (2). To linearize the equation, we took the logarithm of both sides and applied the variable substitution $x=T^{-1}$. This manipulation resulted in the equation taking the form $\ln (D) = \ln (D_0) - E_a/k_B\cdot x$ (Fig. 5). Using the diffusion coefficients obtained from the ReaxFF MD simulations, we fitted a linear model of the form $\ln (D) = \beta _0 + \beta _1\cdot x + \varepsilon$ to obtain the activation energy as function slope of the line, i.e., $E_a = - \beta _1 \cdot k_B$.

To obtain the reported 95% confidence intervals for the diffusivity and activation energy in Table 4, we assumed that the statistical error $\varepsilon$ of the linear model follows a Student’s t-distribution $\mathcal {T}_{\nu }$, where $\nu$ represents the degrees of freedom of the distribution^117^. In our case, $\nu$ equals the number of sampled data *n* minus the constraints of our model, which are the intercept and slope of the model ($\nu =n-2$). Under these reasonable hypotheses, the uncertainty for the diffusivity, $\delta D$, and activation energy, $\delta E_a$, is estimated as follows^117^:

5 $$\begin{aligned} \begin{aligned} \delta D&= t_{n-2,\,0.025} \cdot \dfrac{ \sqrt{ \frac{1}{n-2} \sum _{i=1}^n \left( \langle \textbf{r}^2 \rangle _i - \alpha _0 - \alpha _1 t_i \right) ^2 } }{ 6 \cdot \sqrt{\sum _{j=1}^n \left( t_j- \left\langle t \right\rangle \right) ^2} }, \\ \delta E_a&= t_{n-2,\,0.025} \cdot \dfrac{ \sqrt{ \frac{1}{n-2} \sum _{i=1}^n \left( \ln (D_i) - \beta _0 - \beta _1 \frac{1}{T_i} \right) ^2 } }{ k_{B} \cdot \sqrt{\sum _{j=1}^n \left( \frac{1}{T_j} - \left\langle \frac{1}{T} \right\rangle \right) ^2} }. \end{aligned} \end{aligned}$$

$t_{n-2,\,0.025}$ is the cuts probability 0.025 from the upper tail of Student’s t distribution $\mathcal {T}_{n-2}$, with $n-2$ degrees of freedom. $\alpha _0$, $\alpha _1$, $\beta _0$, and $\beta _1$ are the coefficients of the two linear models determined from the linear regression. $\langle \textbf{r}^2 \rangle _i$ represents the MSD at the *i*-th time $t_i$ of the simulation, $k_B$ is the Boltzmann constant, and $D_i$ and $T_i$ are the diffusivity and corresponding simulation temperature values, respectively. We use the notation $\left\langle \; \cdot \; \right\rangle$ to indicate the mean value of the independent sampled variable of each model, i.e., the time, *t*, for the first model, and the reciprocal of the temperature, $T^{-1}$, for the second. The confidence interval for the entire linear model depicted in Fig. 6 was instead obtained with the Eq. (6), which provides the uncertainty for the logarithm of the diffusivity, $\ln (D)$, as a function of the reciprocal of the temperature, $T^{-1}$, which is the independent variable of the linear model^117^:

6 $$\begin{aligned} \begin{aligned} \delta \left[ \ln (D)\right] \left( \frac{1}{T}\right) =&t_{n-2,\,0.025} \cdot \\&\sqrt{ \frac{1}{n-2} \sum _{i=1}^n \left( \ln (D_i) - \beta _0 - \beta _1 \frac{1}{T_i} \right) ^2 } \cdot \\&\sqrt{ \dfrac{1}{n} + \dfrac{ \left( \frac{1}{T} - \left\langle \frac{1}{T} \right\rangle \right) ^2 }{ \sqrt{\sum _{j=1}^n \left( \frac{1}{T_j} - \left\langle \frac{1}{T} \right\rangle \right) ^2} } }. \end{aligned} \end{aligned}$$

### Supplementary Information

Supplementary Information.

## Data Availability

Furthermore, the energies, forces, settings, and all other calculated properties obtained from DFT simulation are stored as ASE SQLite3 and can be accessed in the repository available at https://github.com/paolodeangelis/Enhancing_ReaxFF_DFT_database or through the Zenodo permalink https://doi.org/10.5281/zenodo.7959121.

## Code availability

In this study, we employed several computational tools to carry out our simulations. We used the open-source LAMMPS code for the ReaxFF MD simulations, while the commercial codes BAND and DFTB were utilized for the DFT and ab initio MD simulations, respectively. These last two codes are all available in the Amsterdam Modeling Suite by SCM. In addition, we employed several Python libraries, including ASE, Pymatgen, and Packmol, to arrange the initial configurations, as well as the PLAMS library and an in-house Python code for automatically managing the simulation campaign. Finally, we utilized the ParAMS library to perform the ReaxFF parametrization. The repository containing all the Jupyter notebooks required for running the ReaxFF reparametrization process, which extensively utilized the aforementioned codes, can be accessed at https://github.com/paolodeangelis/Enhancing_ReaxFF or via Zenodo permalink https://doi.org/10.5281/zenodo.8036775.
