# Supplementary material for: Enhancing ReaxFF for molecular dynamics simulations of lithium-ion batteries: an interactive reparameterization protocol
Source: Sci Rep. 2024 Jan 10;14:978. doi: 10.1038/s41598-023-50978-5 (PMC10782028; doi:10.1038/s41598-023-50978-5)
Supplement: Supplementary file 1 — Supplementary Information. [file 41598_2023_50978_MOESM1_ESM.pdf]

Enhancing ReaxFF for Molecular Dynamics Simulations of Lithium-Ion Batteries: An interactive reparameterization protocol (Supplementary Material)

Paolo De Angelis[1,*], Roberta Cappabianca[1], Matteo Fasano[1], Pietro Asinari[1,2,†], and Eliodoro Chiavazzo[1,‡]

[1]Department of Energy “Galileo Ferraris”, Politecnico di Torino, Corso Duca degli Abruzzi 24, 10129 Torino, Italy.
[2]Istituto Nazionale di Ricerca Metrologica, Strada delle Cacce 91, 10135 Torino, Italy.
[*]paolo.deangelis@polito.it
[†]pietro.asinari@polito.it
[‡]eliodoro.chiavazzo@polito.it

This is the supplementary material for the article “Enhancing ReaxFF for Molecular Dynamics Simulations of Lithium-Ion Batteries: An interactive reparameterization protocol.” Here we list resources and images that aim to provide further explanation and understanding of the methods and results explained in the main text.

In line with the FAIR principles (Findability, Accessibility, Interoperability, and Reusability)[1], we provide detailed documentation within the two repositories (Section 1 and Section 2) that accompany the article. These repositories are designed to help the reproducibility of our results and facilitate further exploration of the LiF configurations or other Solid Electrolytes Interphase (SEI)[2] compounds, extending the database and improving the ReaxFF force field[3].

Contents

1 Enhancing ReaxFF protocol (Protocol Repository) 2
1.1 Installation . . . 2
Minimum Requirements • Requirements for Re-running or Performing New Simulations • Requirements for Re-running ReaxFF optimization • Python project setup
1.2 Folder Structure . . . 4
2 Enhancing ReaxFF database (Database Repository) 6
2.1 Installation . . . 9
Minimum Requirements • Requirements for Re-running or Performing New Simulations • Python project setup
2.2 Interacting with the Database . . . 9
ASE db Command-line • ASE Python Interface • Web Interface
2.3 Folder Structure . . . 12
3 DFT simulations 14
3.1 DFT vs. ReaxFF mechanical deformation . . . 18
4 Diffusion MSD analysis 18
5 Reparameterized ReaxFF 21
References 24

1 Enhancing ReaxFF protocol (Protocol Repository)

In this repository, we collect and organize all the steps described in the main text for reparameterizing the ReaxFF potential. To facilitate the interaction with the protocol, we have divided the workflow, illustrated in Figure S1, into four main Jupyter Notebooks (JNBs) and an auxiliary notebook: `JNB1-Initial_configurations.ipynb`, `JNB2-Simulations.ipynb`, `JNB3-Build_trainingset.ipynb`, `preJNB4-ReaxFF_optimization.ipynb`, `JNB4-ReaxFF_optimization.ipynb`. These provided

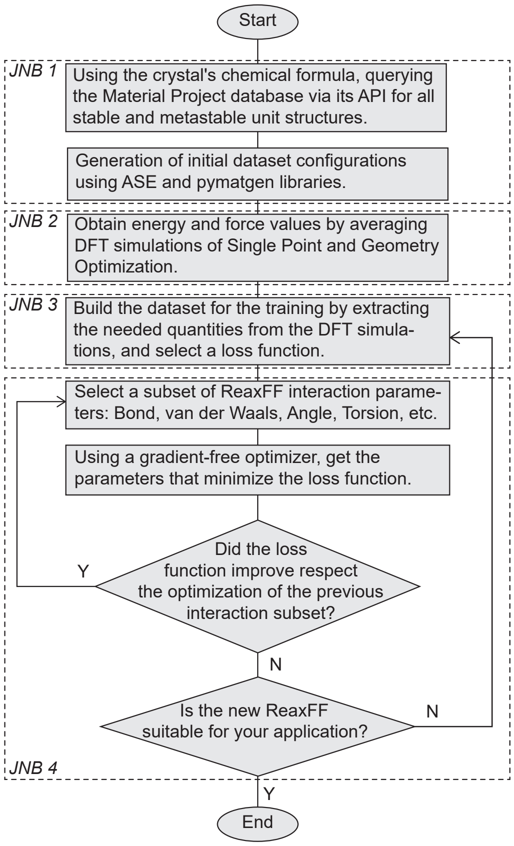

Figure S1. Protocol workflow overview

Jupyter Notebooks are specifically designed to streamline the process of configuration building (JNB1), DFT simulation execution (JNB2), database preparation (JNB3), and ReaxFF potential optimization (JNB4), as outlined in the workflow in Figure S1. The conditional part of the workflow is achieved using the fourth and auxiliary notebook since the auxiliary notebook (`preJNB4-ReaxFF_optimization.ipynb`) is selected the subset of ReaxFF parameters related to a specific interaction and then fed to the JNB4, which will change it to minimize the loss function.

The repository is available both in Zenodo at the permanent link `https://doi.org/10.5281/zenodo.8036775`, and on version control repositories host GitHub `https://github.com/paolodeangelis/Enhancing_ReaxFF`, Figure S2.

1.1 Installation

The protocol strongly relies on the commercial code Amsterdam Modeling Suite (AMS) by Software for Chemistry & Materials (SCM)[4]. However, depending on which part of the protocol you aim to reproduce, it is possible to install only the minimal requirements, as described in Subsubsection 1.1.1, which allow for the generation and handling of atomistic simulations. To perform the simulations, additional requirements outlined in Subsubsection 1.1.2 are necessary. For the ReaxFF optimization, the Python library ParAMS[5] is indispensable, and it is currently available

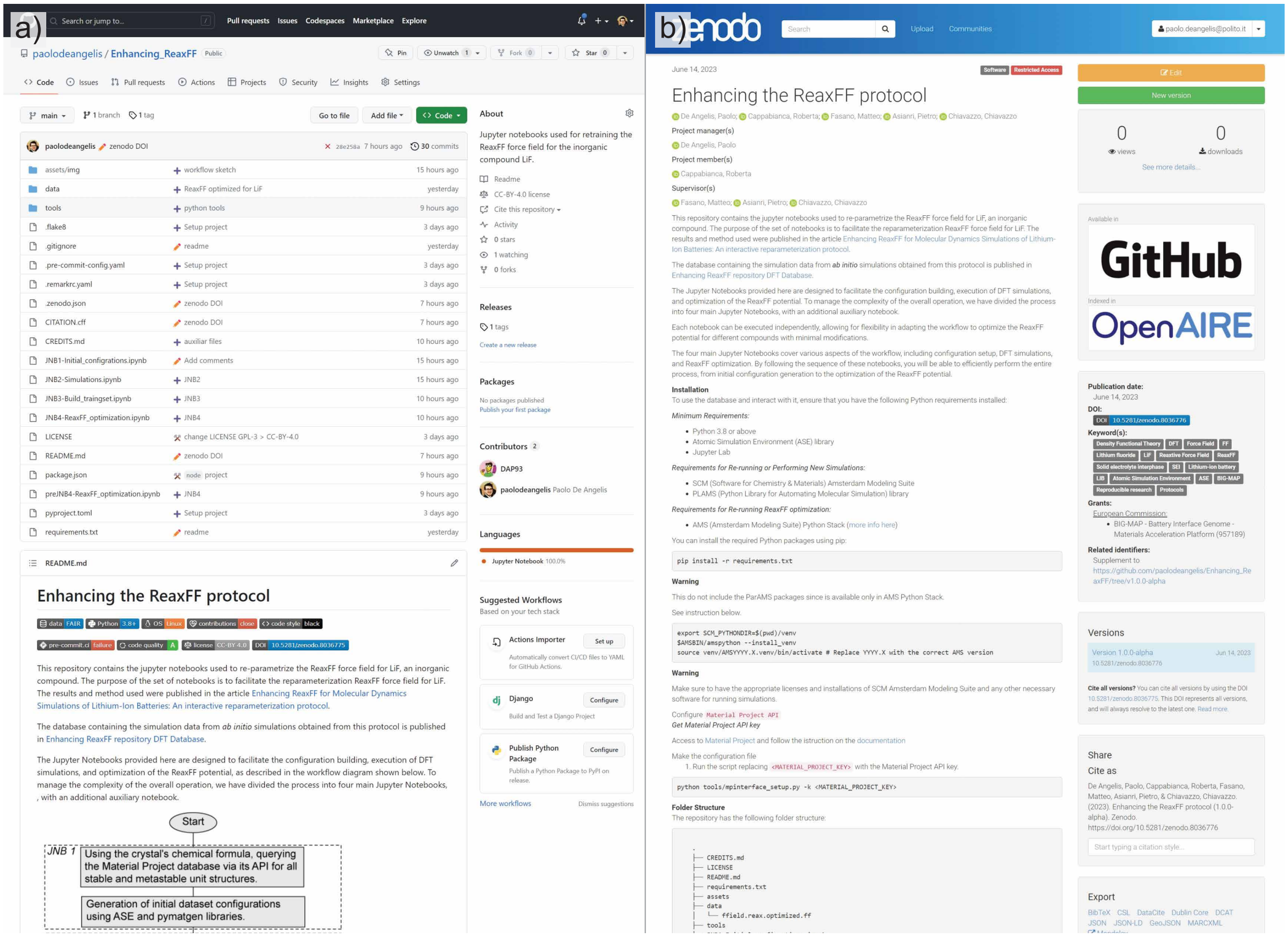

Figure S2. Screenshot of the protocol hosted on GitHub (a) and Zenodo (b).

only within the AMS Python Stack, as explained in Subsubsection 1.1.4.

1.1.1 Minimum Requirements

- Python 3.8 or above
- Atomic Simulation Environment (ASE) library
- Jupyter Lab

1.1.2 Requirements for Re-running or Performing New Simulations

- SCM (Software for Chemistry & Materials) Amsterdam Modeling Suite
- PLAMS (Python Library for Automating Molecular Simulation) library

1.1.3 Requirements for Re-running ReaxFF optimization

- AMS (Amsterdam Modeling Suite) Python Stack (more info here). To utilize the AMS Python Stack for this project, we recommend creating a Python virtual environment as follow:

```
export SCM_PYTHONDIR=$(pwd)/venv
$AMSBIN/amspython --install_venv
source venv/AMSYYYY.X.venv/bin/activate # Replace YYYY.X with the correct AMS
    version
```

1.1.4 Python project setup

Regardless of the software available, the next step is to install all the additional Python packages (such as pymatgen, ASE, etc.) required for the protocol. This can be accomplished using the Python package manager, `pip`, through the following terminal command:

```
(venv)$ pip install -r requirements.txt
```

Then it is necessary to set up the Material Project "API keys" to enable the code to query the database. To obtain the API key, follow the instructions provided in the documentation, which can be found here. After obtaining the API key, replace `<MATERIAL_PROJECT_KEY>` with your actual API key and run the script as shown below:

```
(venv)$ python tools/mpinterface_setup.py -k <MATERIAL_PROJECT_KEY>
```

Finally, to utilize the Jupyter Notebooks, it is necessary to run the Jupyter Lab server using the following command:

```
(venv)$ jupyter lab
```

1.2 Folder Structure

The repository is structured as follows:

```
.
├── assets
├── data
│   └── ffield.reax.optimized.ff
├── tools
│   └── ...
├── ...
├── CREDITS.md
├── LICENSE
├── README.md
├── requirements.txt
├── JNB1-Initial_configrations.ipynb
├── JNB2-Simulations.ipynb
├── JNB3-Build_traingset.ipynb
├── preJNB4-ReaxFF_optimization.ipynb
└── JNB4-ReaxFF_optimization.ipynb
```

In the folder tree, we have excluded certain auxiliary files and folders that are not essential for understanding the repository, and below we explain in detail the content of each folder/file:

- `assets`: This folder contains any additional assets, such as images or documentation; related to the repository.
- `data`: This folder contains the data files resulting from this work.
  - `ffield.reax.optimized.ff`: This file is the optimized ReaxFF resulting from using these Jupyter notebooks, as explained in the main text, and as reported in Section 5.

- `tools`: This directory contains a collection of Python modules and scripts.
- `CREDITS.md`: This file acknowledges and credits each author who contributed to the repository.
- `LICENSE`: This file contains the license information for the repository (CC BY 4.0). It specifies the terms and conditions under which the repository's contents are distributed and used.
- `README.md`: This is the "readme" file (repository overview and instructions).
- `requirements.txt`: This file lists the required Python packages and their versions (see Subsection 1.1).
- `JNB1-Initial_configurations.ipynb`: Jupyter Notebook where the protocol is initialized by querying the *Materials Project* database, downloading the unit crystals, and producing all the initial configurations for the DFT simulations using the *pymatgen* library.
- `JNB2-Simulations.ipynb`: This notebook performs the simulations using BAND and DFTB codes available in the Amsterdam Modeling Suite. The simulations are performed in parallel using the *PLAMS* library and *SLURM* scheduler.
- `JNB3-Build_trainingset.ipynb`: Here, the quantities needed for the database are extracted and tuned to favor accuracy on the energy.
- `preJNB4-ReaxFF_optimization.ipynb`: This is an auxiliary Notebook where the old ReaxFF is converted into a Python object, and it is possible to select the subset of coefficients related to specific interactions to change during the optimization (e.g., bond, van der Waals, angular, etc.).
- `JNB4-ReaxFF_optimization.ipynb`: This notebook takes the database and the ReaxFF Python object to perform a multi-objective optimization and find the new ReaxFF potential that minimizes the Sum of Squared Errors (SSE).

2 Enhancing ReaxFF database (Database Repository)

In addition to the protocol, we are sharing the complete database used to reparameterize ReaxFF. This allows for testing the optimization without rerunning time-consuming simulations and enables future expansion and improvement by the community. We stored the data using the ASE SQLite3 database, which was chosen for its widespread use in the computational community of European institutions. The use of this type database provides fast and flexible querying and browsing capabilities, as described in Subsection 2.2. The database contains information on initial configurations, single-point calculations, pre-optimizations, and DFT optimizations. However, only a subset of the database is used in the training set for the ReaxFF optimization, as explained in the main text and briefly summarized in Table S1. For each entry in the database, we store various information regarding the simulation and

Table S1. Training set composition, with quantities calculated using DFT: Energy (E), Forces (F), and Charges (Q). The numbers in parentheses indicate the successful cases for self-consistent field (SCF) calculations that may not always converge.

**Type**	**Unit cell**			**Quantities**		
	$Fm\bar{3}m$	$P6_3mc$	$Pm\bar{3}m$	E	F	Q
Supercells	6	6	6	✓	✓	✓
Vacancies	10	24	10	✓		✓
Strain	39	39	39	✓		✓
Substitution	5	5	5	✓		✓
Interstitial	5	5	5	✓	✓	✓
Slabs	12(10)	12(8)	12(11)	✓	✓	✓
Bulk 300 K	10	10	10	✓		
Bulk 500 K	10	10	10	✓		
Amorphous	10	10	10	✓		
Total	107(105)	121(117)	107(106)			

properties obtained from the DFT calculations. These details are listed in Table S2.

The repository is available both in Zenodo at the permanent link `https://doi.org/10.5281/zenodo.7959121`, and on version control repositories host GitHub `https://github.com/paolodeangelis/Enhancing_ReaxFF_DFT_database`, Figure S3.

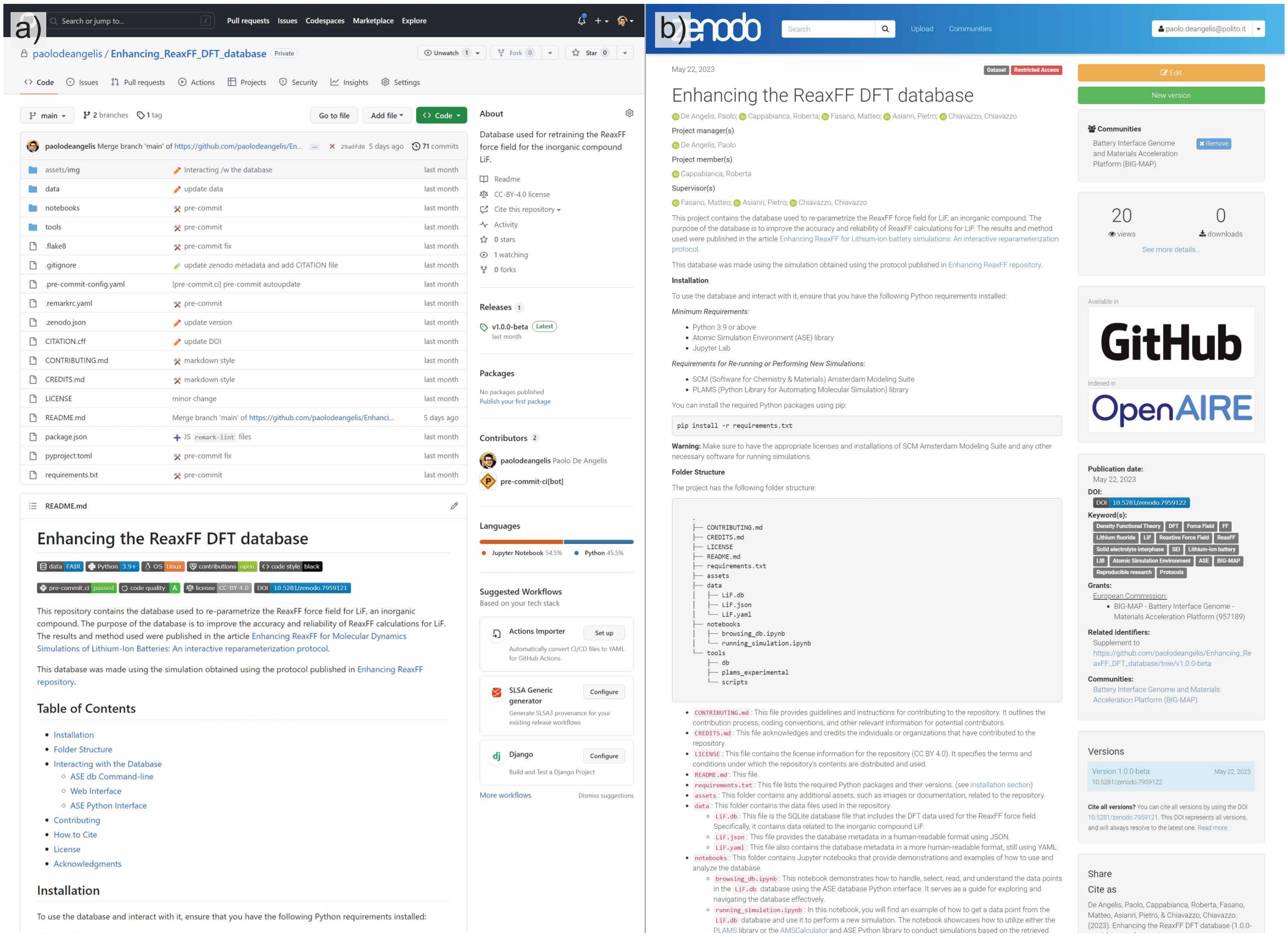

Figure S3. Screenshot of the database hosted on GitHub (a) and Zenodo (b).

Table S2. Datailed list of properties and information store in each database entry

name	description	type	unit
id	Uniqe row ID	integer	–
ctime	Creation time of the data (for simulation, it coincides with the runtime)	float	yr
formula	Chemical formula of the system	string	–
pbc	Periodic boundary conditions	boolean	–
user	Username or full name of the user who created the data	string	–
calculator	Name of the ASE-calculator and engine used to calculate the system (e.g ams/band means AMS calculator with BAND engine)	string	–
energy	Total energy of the system from the calculation	float	eV
natoms	Number of atoms	integer	–
fmax	Maximum force	float	$\mathrm{eV\,\AA^{-1}}$
smax	Maximum stress	float	$\mathrm{eV/\AA^{3}}$
charge	Net charge in unit cell	float	$\vert e \vert$
mass	Sum of atomic masses in unit cell	float	au
magmom	Magnetic moment	float	μ_B
unique_id	Random (unique) ID	integer	–
volume	Volume of unit cell	float	$\mathrm{\AA^{3}}$
functional	Exchange-and-correlation functional	string	–
fermi_energy	Fermi Energy (N.B. is not the Fermi Level), which indicates the energy of non-interacting fermions in the system (Fermi gas) at 0 K	float	eV
homo_energy	Highest Occupied Molecular Orbital energy	float	eV
lumo_energy	Lowest Unoccupied Molecular Orbital energy	float	eV
band_gap	Band gap energy (for LiF is the HOMO energy - LUMO energy)	float	eV
runtime	Simulation start date whit format %b%d-%Y %H:%M:%S	string	–
elapsed	Elapsed Time	float	s
name	System Name	string	–
sim_name	Simulation Name	string	–
subset_name	Name of the subset of configuration	string	–
run_script	Full AMS scrip for running the simulation	string	–
input_script	Full input scrip for running the simulation	string	–
success	Simulation end status	boolean	–
used_in	Indicate in which set (training-set or test-set) the data was used	string	–
task	Simulation task type	string	–
space_group	Full International Space Group Symbol. The notation is a LaTeX-like string, with screw axes being represented by an underscore	string	–
data	Additional data calculate: Density of states(DOS) and Hystroy (e.g. energy, force evolution during the simulation)	dictionary	–

2.1 Installation

To browse and query the database, only Python and the ASE and Jupyter Lab libraries are required (see Subsubsection 2.1.1). With this minimal setup, it is possible to perform additional simulations using the preferred DFT engine. Using ASE, you can wrap the simulations and add them to the database. For an example, you can refer to the `notebooks\running_simulation.ipynb` Jupyter Notebook, which demonstrates the usage of the BAND plane-wave DFT code. However, if you wish to rerun the simulations stored in the database, the commercial code Amsterdam Modeling Suite (AMS) is required (refer to Subsubsection 2.1.2).

2.1.1 Minimum Requirements

- Python 3.8 or above
- Atomic Simulation Environment (ASE) library
- Jupyter Lab

2.1.2 Requirements for Re-running or Performing New Simulations

- SCM (Software for Chemistry & Materials) Amsterdam Modeling Suite
- PLAMS (Python Library for Automating Molecular Simulation) library

2.1.3 Python project setup

Similar to the protocol repository, the project setup requires the installation of additional Python packages such as ASE, PLAMS, etc. These packages can be installed using the Python package manager, `pip`, with the following terminal command:

```
(venv)$ pip install -r requirements.txt
```

Then it is possible to utilize the Jupyter Notebooks by starting the Jupyter Lab server using the following command:

```
(venv)$ jupyter lab
```

2.2 Interacting with the Database

There are three methods available for interacting with the database: using the ASE db command line, the web interface, and the ASE Python interface.

2.2.1 ASE db Command-line

To interact with the database through the ASE db command line, follow these steps:

1. Open a terminal and navigate to the directory where the `LiF.db` file is located.
2. Execute the following command to initiate the ASE db terminal:

   ```
   (venv)$  ase db LiF.db
   ```

3. Now, one can utilize the available commands within the terminal to query and manipulate the database.

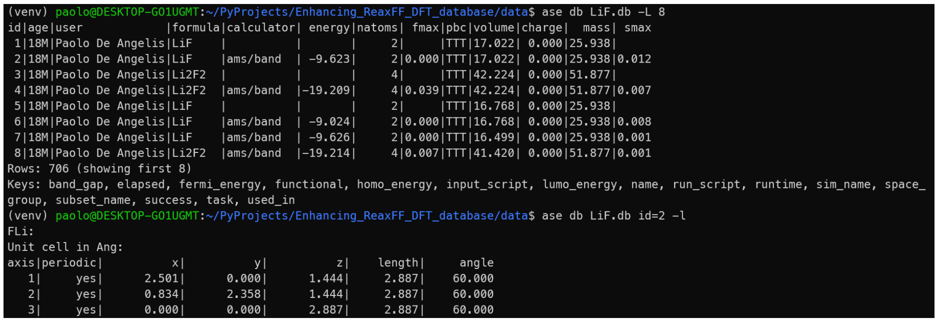

```
(venv) paolo@DESKTOP-GO1UGMT:~/PyProjects/Enhancing_ReaxFF_DFT_database/data$ ase db LiF.db -L 8
id|age|user           |formula|calculator| energy|natoms| fmax|pbc|volume|charge|  mass| smax
 1|18M|Paolo De Angelis|LiF    |          |        |     2|     |TTT|17.022| 0.000|25.938|
 2|18M|Paolo De Angelis|LiF    |ams/band  | -9.623|     2|0.000|TTT|17.022| 0.000|25.938|0.012
 3|18M|Paolo De Angelis|Li2F2  |          |        |     4|     |TTT|42.224| 0.000|51.877|
 4|18M|Paolo De Angelis|Li2F2  |ams/band  |-19.209|     4|0.039|TTT|42.224| 0.000|51.877|0.007
 5|18M|Paolo De Angelis|LiF    |          |        |     2|     |TTT|16.768| 0.000|25.938|
 6|18M|Paolo De Angelis|LiF    |ams/band  | -9.024|     2|0.000|TTT|16.768| 0.000|25.938|0.008
 7|18M|Paolo De Angelis|LiF    |ams/band  | -9.626|     2|0.000|TTT|16.499| 0.000|25.938|0.001
 8|18M|Paolo De Angelis|Li2F2  |ams/band  |-19.214|     4|0.007|TTT|41.420| 0.000|51.877|0.001
Rows: 706 (showing first 8)
Keys: band_gap, elapsed, fermi_energy, functional, homo_energy, input_script, lumo_energy, name, run_script, runtime, sim_name, space_
group, subset_name, success, task, used_in
(venv) paolo@DESKTOP-GO1UGMT:~/PyProjects/Enhancing_ReaxFF_DFT_database/data$ ase db LiF.db id=2 -l
FLi:
Unit cell in Ang:
axis|periodic|          x|          y|          z|    length|     angle
   1|     yes|      2.501|      0.000|      1.444|     2.887|    60.000
   2|     yes|      0.834|      2.358|      1.444|     2.887|    60.000
   3|     yes|      0.000|      0.000|      2.887|     2.887|    60.000
```

Figure S4. Example of interacting with the database via the terminal.

2.2.2 ASE Python Interface

To interact with the database using the ASE Python interface, you can utilize the following code example:

```python
from ase.db import connect

# Connect to the database
db = connect("LiF.db")

# Query the database
results = db.select('success=True')

# Iterate over the results
for row in results:
    print(f"ID: {row.id}, Energy: {row.energy}")
```

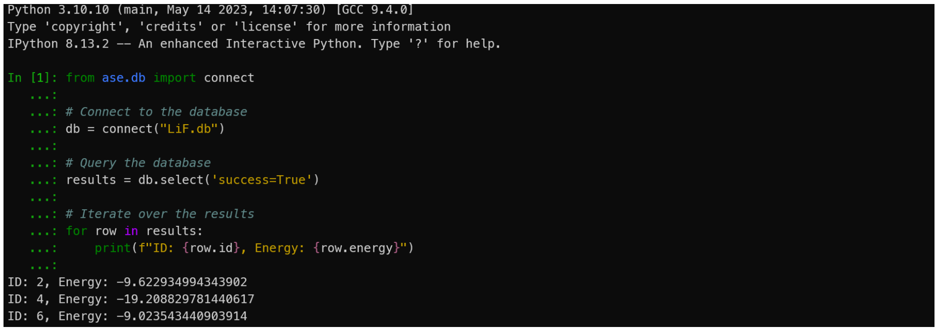

Figure S5. Example of interacting with the database via Python, utilizing the iPython terminal interface.

For a more detailed example, refer to the notebook `notebooks\browsing_db.ipynb`. To learn how to perform a simulation, check the notebook `notebooks\running_simulation.ipynb`.

2.2.3 Web Interface

To interact with the database using the web interface, follow these steps:

1. Open a terminal and navigate to the directory where the `LiF.db` file is located.
2. Execute the following command to initiate the ASE db terminal:

```
(venv)$ ase db -w LiF.db
```

3. Access the web browser and connect to the local server at `http://127.0.0.1:5000`.
4. Upon accessing the interface, Figure S6.a will be displayed, showcasing all the entries in the database.
5. To query the database and filter the entries based on specific criteria, users can input their search parameters into the designated fields (e.g. `success=True`).
6. Once the query is executed, Figure S6.b will be displayed, presenting the selected entries that match the search criteria.
7. To access all the information of an individual entry, users can click on a specific entry to view a detailed visualization. Figure S7 demonstrates an example of a single entry displayed through the web interface.

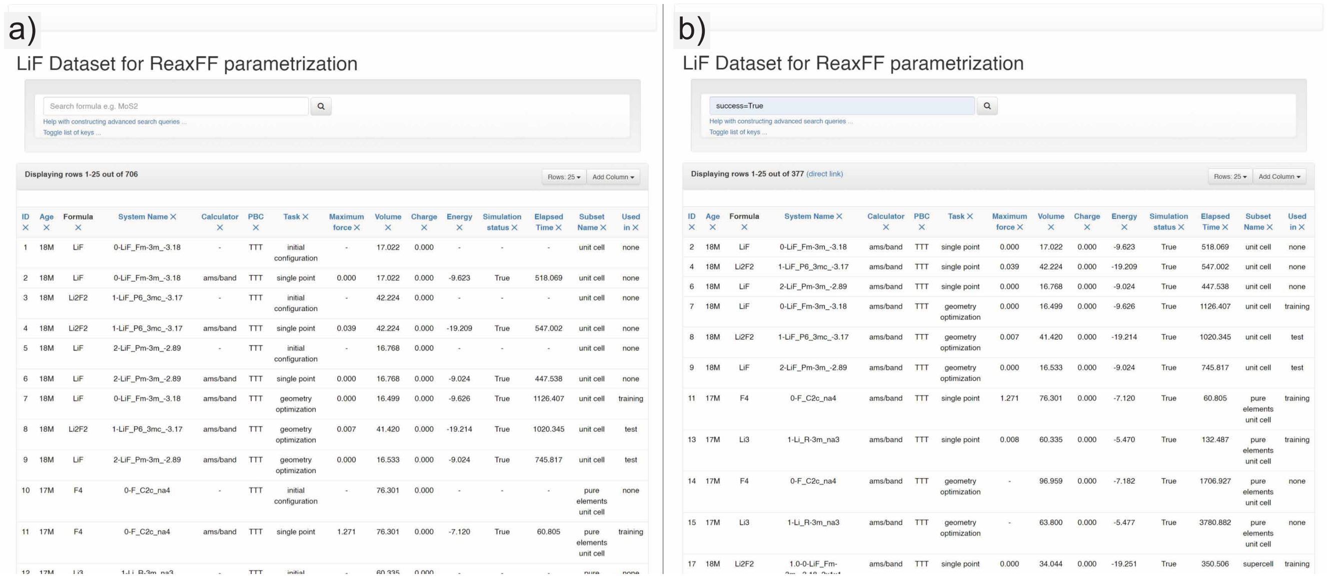

Figure S6. Screenshot showing the web interface of the database, displaying all the entries (a), and filtering the entries after querying the database (b).

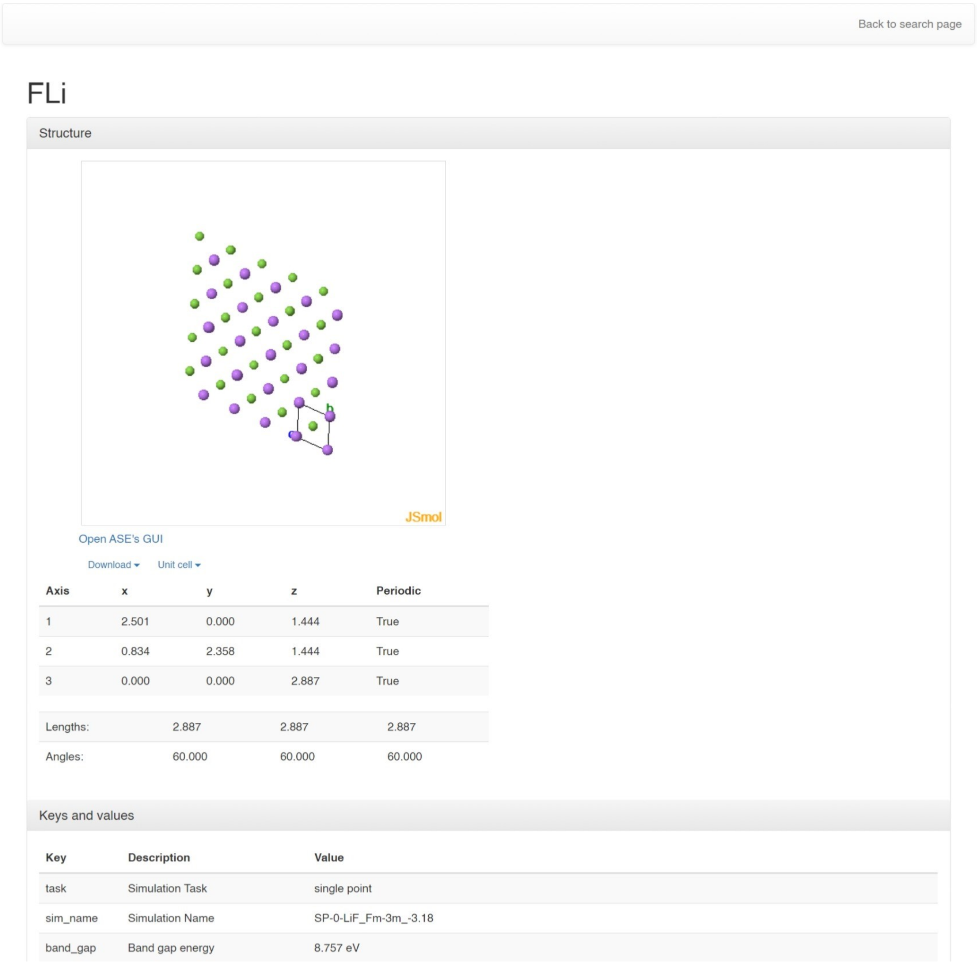

Figure S7. Screenshot displaying a entrie from the database visualized using the web interface.

2.3 Folder Structure

The repository is structured as follows:

```
.
├── assets
├── data
│   ├── LiF.db
│   ├── LiF.json
│   └── LiF.yaml
├── notebooks
│   ├── browsing_db.ipynb
│   └── running_simulation.ipynb
├── tools
│   ├── db
│   ├── plams_experimental
│   └── scripts
├── ...
├── CONTRIBUTING.md
├── CREDITS.md
├── LICENSE
├── README.md
└── requirements.txt
```

- `assets`: This folder contains any additional assets, such as images or documentation, related to the repository.
- `data`: This folder contains the data files used in the repository.
  - `LiF.db`: This file is the SQLite database file that includes the DFT data used for the ReaxFF force field. Specifically, it contains data related to the inorganic compound LiF.
  - `LiF.json`: This file provides the database metadata in a human-readable format JSON.
  - `LiF.yaml`: This file also contains the database metadata in a more human-readable format YAML.
- `notebooks`: This folder contains Jupyter notebooks that provide demonstrations and examples of how to use and analyze the database.
  - `browsing_db.ipynb`: This notebook demonstrates how to handle, select, read, and understand the data points in the `LiF.db` database using the ASE database Python interface. It serves as a guide for exploring and navigating the database effectively.

  - running_simulation.ipynb: In this notebook, you will find an example of how to get a data point from the LiF.db database and use it to perform a new simulation. The notebook showcases how to utilize either the PLAMS library or the AMSCalculator and ASE Python library to conduct simulations based on the retrieved data and then store it as a new data point in the LiF.db database. It provides step-by-step instructions and code snippets for a seamless simulation workflow.

- tools: This directory contains a collection of Python modules and scripts that are useful for reading, analyzing, and re-running simulations stored in the database. These tools are indispensable for ensuring that this repository adheres to the principles of **I**nteroperability and **R**eusability, as outlined by the FAIR principles.

  - db: This Python module provides functionalities for handling, reading, and storing data into the database.

  - plasm_experimental: This Python module includes the necessary components for using the AMSCalculator with PLASM and the SCM software package, utilizing the ASE API. It facilitates running simulations, and performing calculations.

  - scripts: This directory contains additional scripts for advanced usage scenarios of this repository.

- CONTRIBUTING.md: This file provides guidelines and instructions for contributing to the repository. It outlines the contribution process, coding conventions, and other relevant information for potential contributors.

- CREDITS.md: This file acknowledges and credits each author who contributed to the repository.

- LICENSE: This file contains the license information for the repository (CC BY 4.0). It specifies the terms and conditions under which the repository's contents are distributed and used.

- README.md: This is the "readme" file (repository overview and instructions).

- requirements.txt: This file lists the required Python packages and their versions. (see Subsection 2.1)

3 DFT simulations

In this section, we present the results of our DFT simulations, highlighting the energy obtained for various configurations.

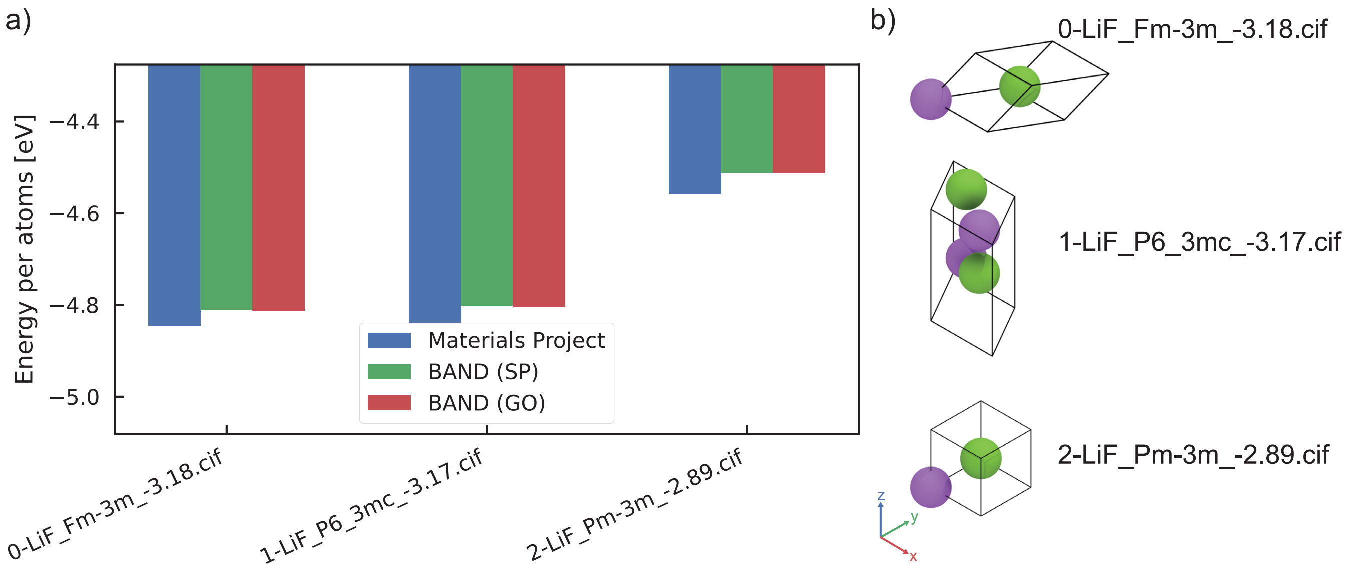

Figure S8. Comparison of energy per atom obtained from DFT simulations (SP: Single Point, GO: Geometry Optimization) with those stored on Material Project (a), for each primitive unit cell of LiF (b).

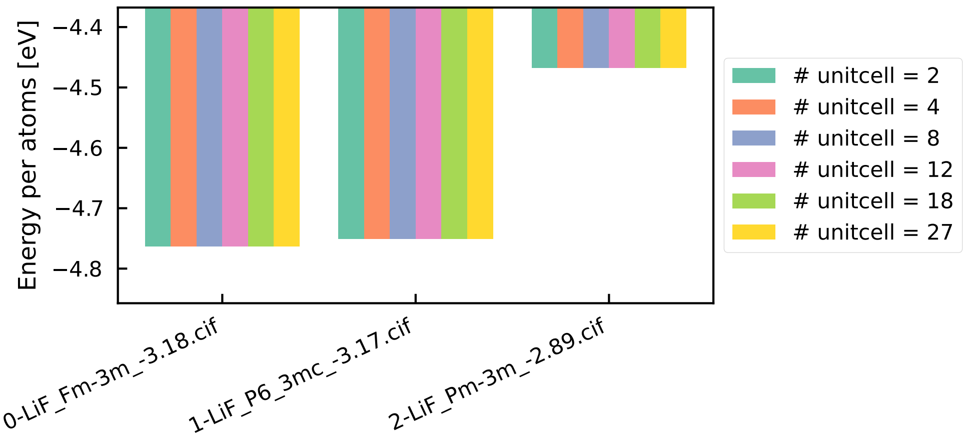

Figure S9. Energy per atom obtained from DFT simulations for each crystal in the LiF supercell.

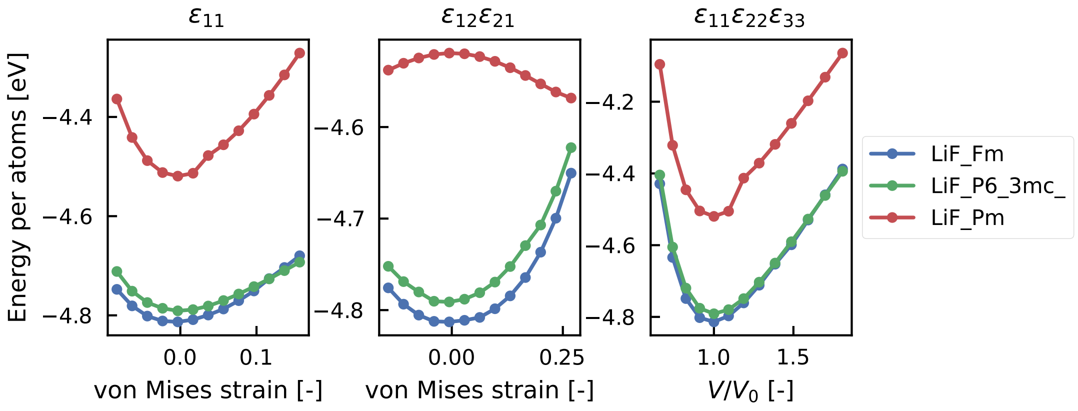

Figure S10. Energy per atom curves obtained from strained configurations, specifically with 1D strain ε_{11}, shear strain $\varepsilon_{12} = \varepsilon_{21}$, and homogeneous expansion/compression $\varepsilon_{11} = \varepsilon_{22} = \varepsilon_{33}$.

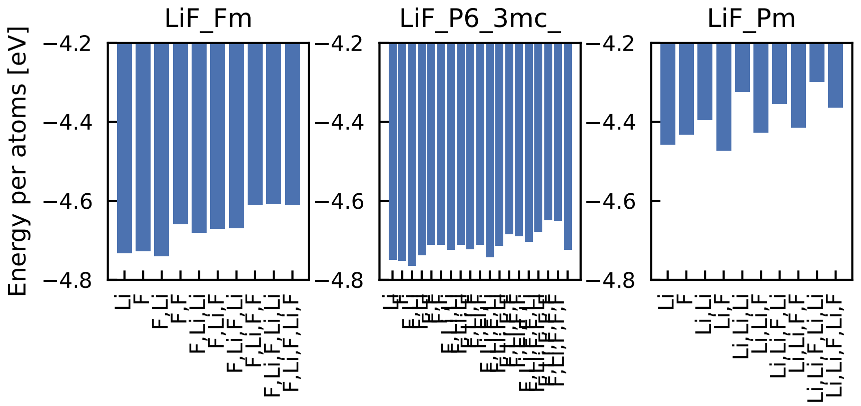

Figure S11. Energy per atom obtained from DFT simulations for each system where a vacancy defect was added.

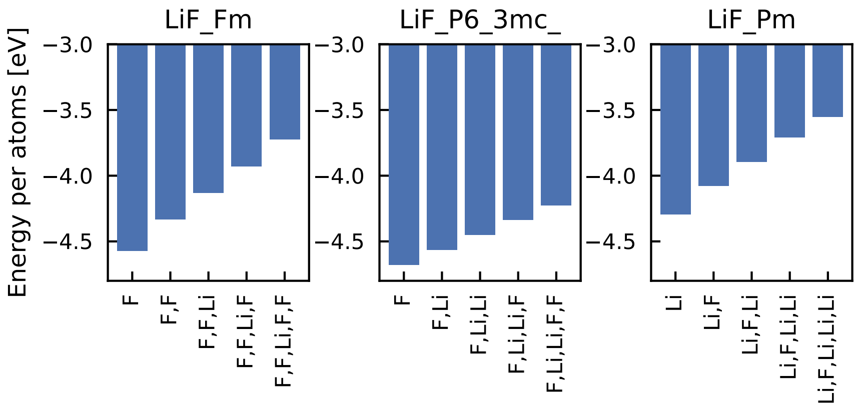

Figure S12. Energy per atom obtained from DFT simulations for each system where a substitution defect was added.

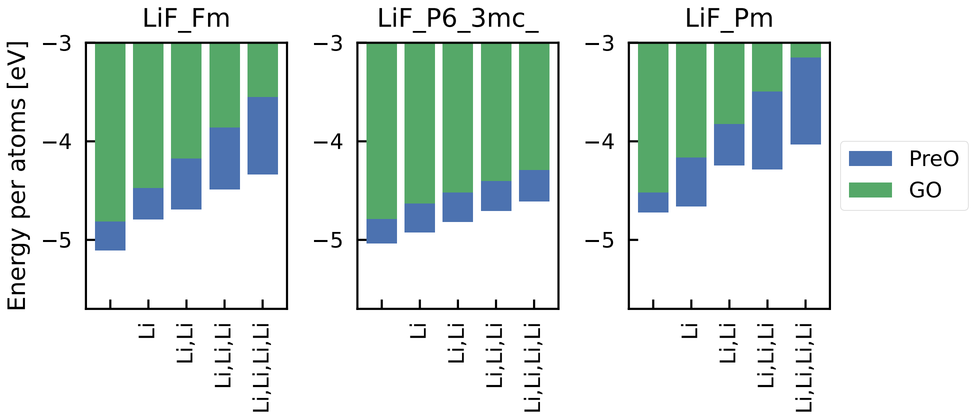

Figure S13. Energy per atom obtained from DFT simulations for each system with an added interstitial defect. Due to the significant crystal deformation introduced, the optimization process was conducted in two steps: Pre-optimization (blue) and full geometry optimization (green).

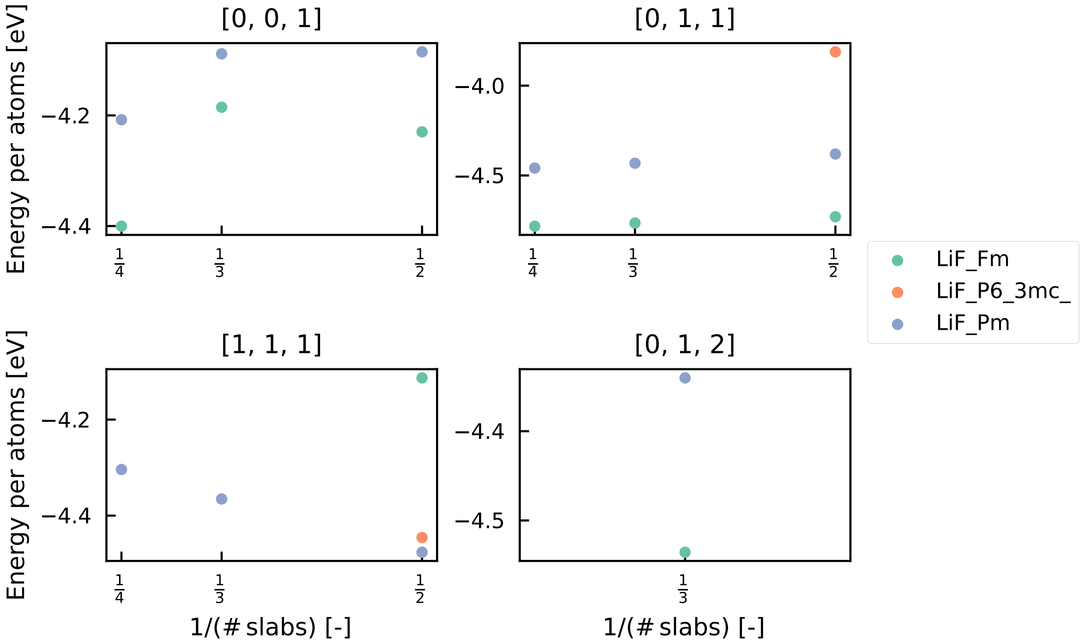

Figure S14. Comparing energy per atom for different crystal plates ($[0,0,1]$, $[0,1,1]$, $[1,1,1]$, $[0,1,2]$) with varying slab thickness. The plot illustrates how the energy per atom changes as a function of the inverse of the number of slabs, emphasizing the impact of surface energy.

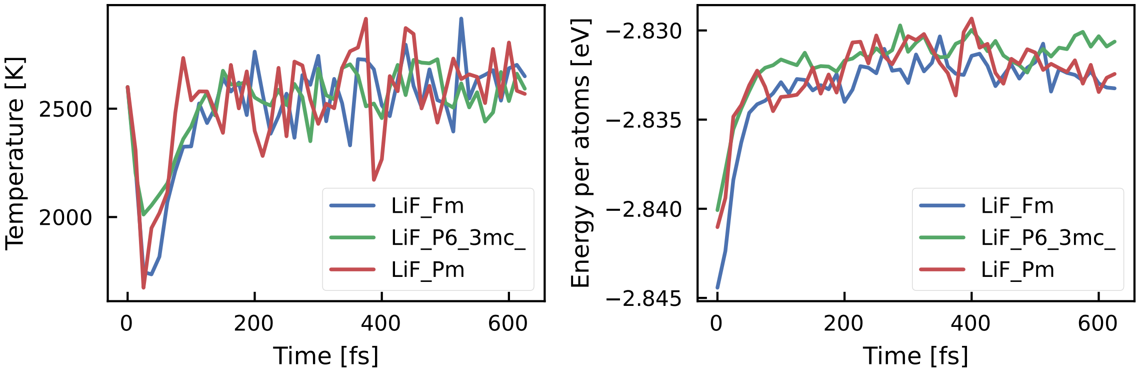

Figure S15. Evolution of temperature and energy per atom in *ab initio* NVT-MD simulation at 2500 K with Non-Scc-GFN1xTB DFTB model[6] for sampling amorphous LiF systems.

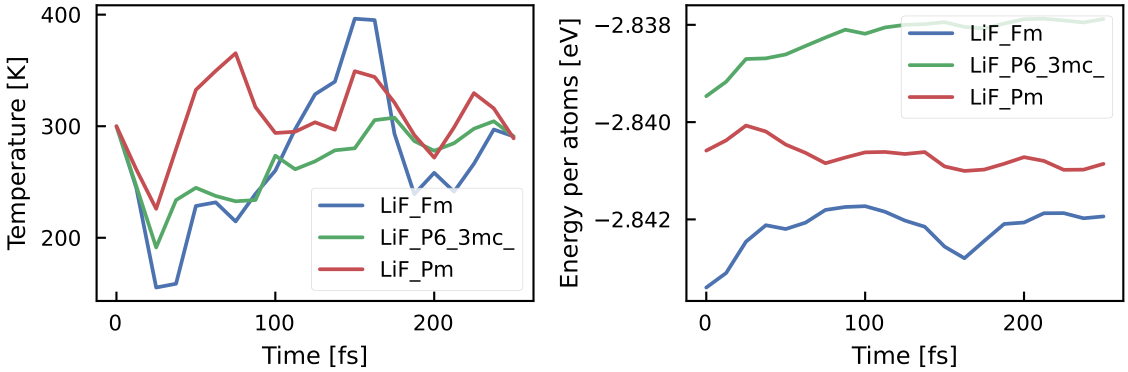

Figure S16. Evolution of temperature and energy per atom in *ab initio* NVT-MD simulation at 300 K with Non-Scc-GFN1xTB DFTB model[6] for sampling solid LiF systems.

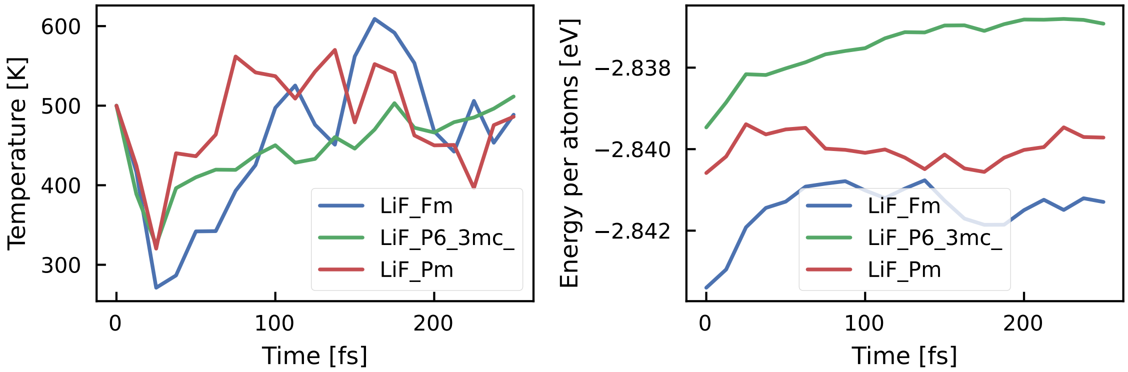

Figure S17. Evolution of temperature and energy per atom in *ab initio* NVT-MD simulation at 500 K with Non-Scc-GFN1xTB DFTB model[6] for sampling solid LiF systems.

3.1 DFT vs. ReaxFF mechanical deformation

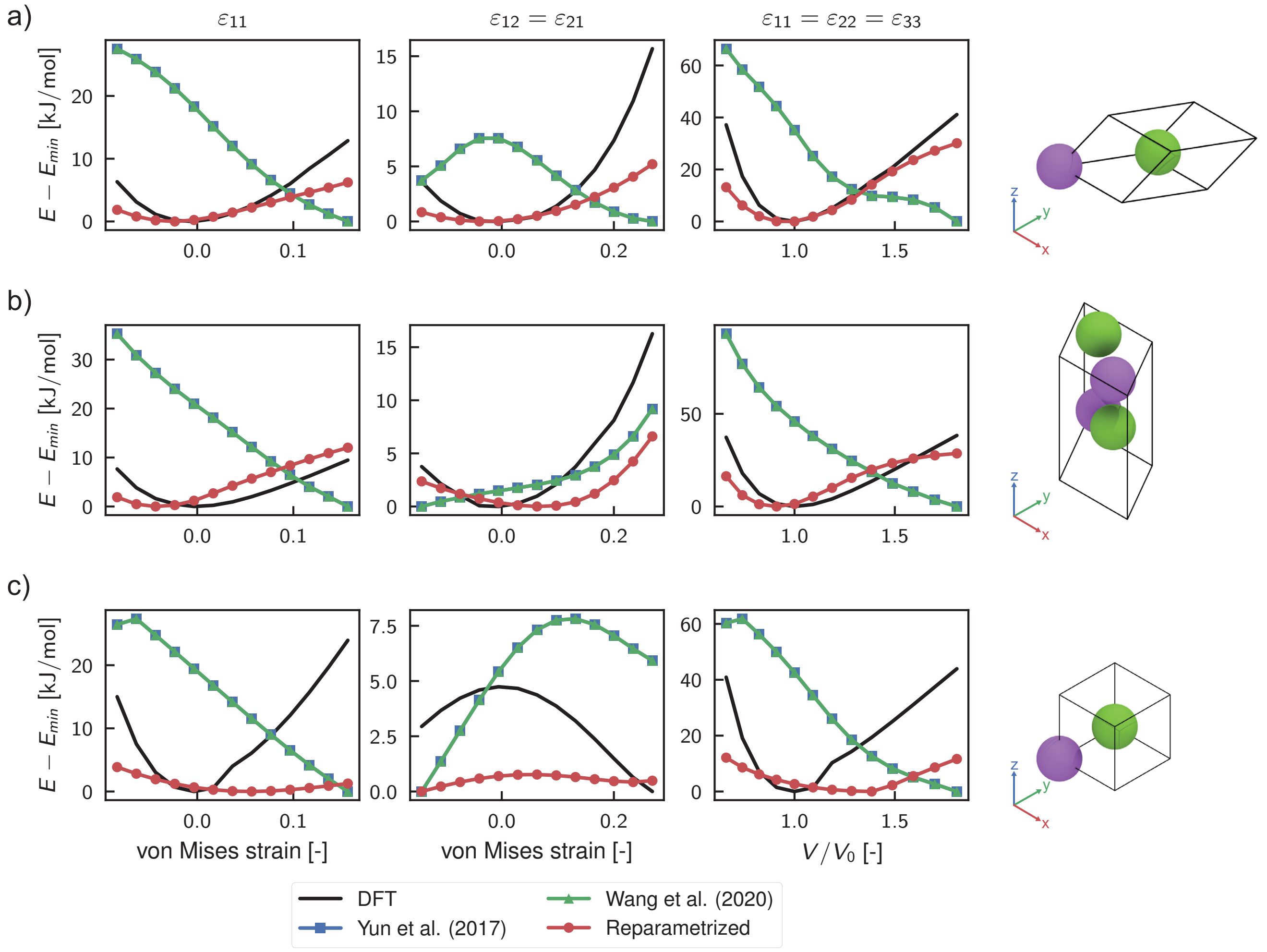

Figure S18. Comparing the mechanical response of LiF: ReaxFF and DFT Predictions. The black line represents DFT results used for training, while the blue, green, and red lines depict the energy predictions by ReaxFF models from Yun et al.[7], Wang et al.[8], and our proposed new reparameterization, respectively. We examine the energy variation with respect to the equilibrium crystal for three types of deformation: tensile strain deformation ε_{11} (left), shear strain deformation $\varepsilon_{12} = \varepsilon_{21}$ (center), and homogeneous lattice length change V/V_0 (where V_0 is the equilibrium volume). This study was performed on all stable and metastable crystals with space groups Fm$\bar{3}$m (a), Pm$\bar{3}$m (b), and P6_3mc (c) from the Material Project database[9].

4 Diffusion MSD analysis

Presented here is a plot illustrating the Mean Square Displacement (MSD) analysis conducted to investigate Li diffusion using ReaxFF simulatios.

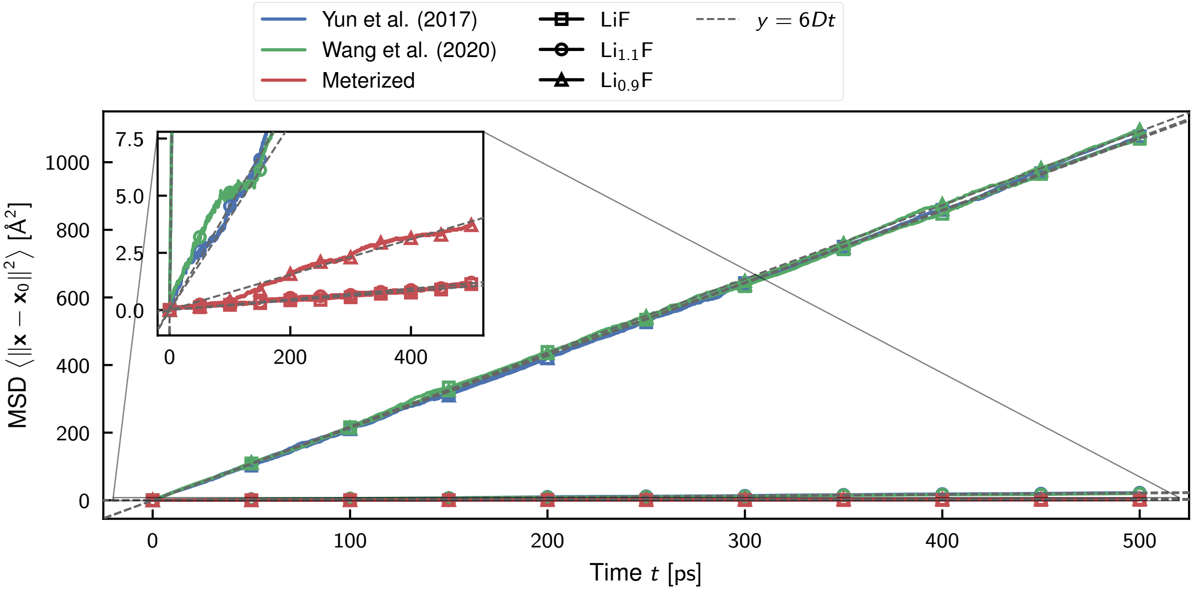

Figure S19. Evolution of Mean Square Displacement (MSD) during NVT-MD ReaxFF simulations of Pure Bulk LiF (LiF) and point defects: vacancy ($Li_{0.9}F$) and interstitial ($Li_{1.1}F$) at 300 K. The markers (square, circle, and triangular) represent the corresponding systems in the plot. The simulations were performed using ReaxFF parameterizations by Yun et al.[7] (blue), Wang et al.[8] (green), and our proposed new parameterization (red). The dashed line indicates the linear model ($MSD = 6D \cdot t$) employed to compute diffusivity in each simulation.

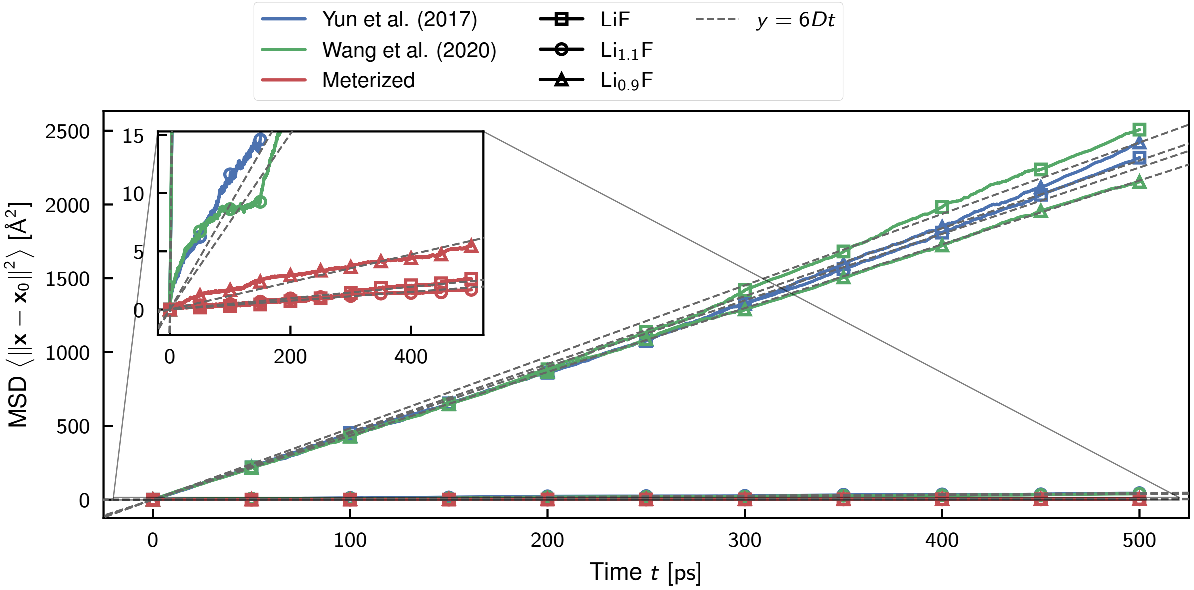

Figure S20. Evolution of Mean Square Displacement (MSD) during NVT-MD ReaxFF simulations of Pure Bulk LiF (LiF) and point defects: vacancy ($Li_{0.9}F$) and interstitial ($Li_{1.1}F$) at 400 K. The markers (square, circle, and triangular) represent the corresponding systems in the plot. The simulations were performed using ReaxFF parameterizations by Yun et al.[7] (blue), Wang et al.[8] (green), and our proposed new parameterization (red). The dashed line indicates the linear model ($MSD = 6D \cdot t$) employed to compute diffusivity in each simulation.

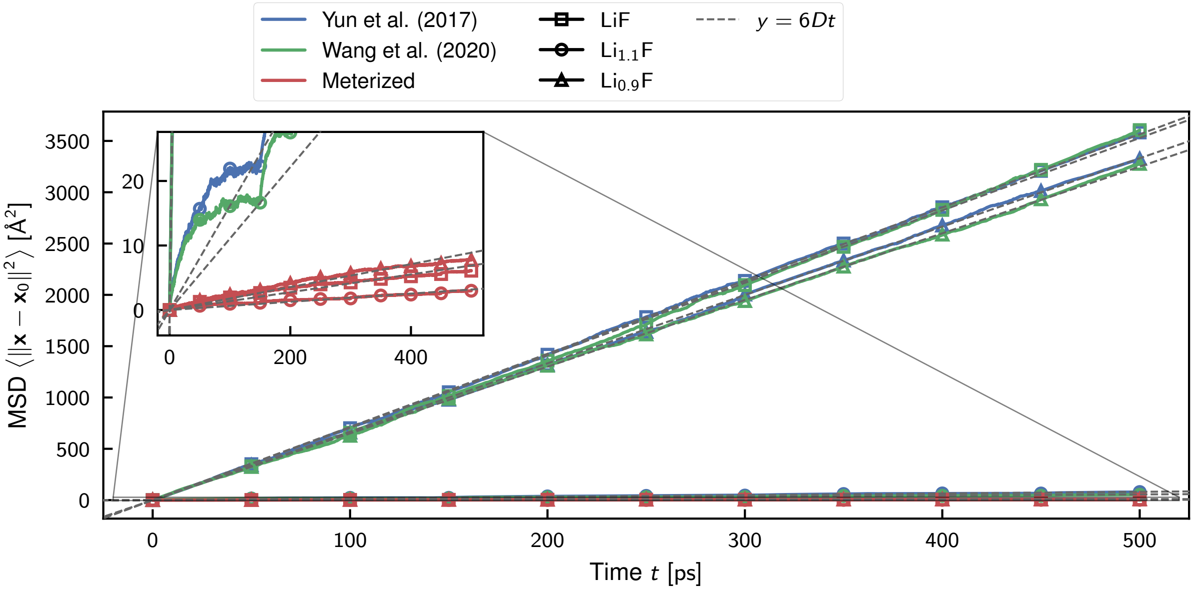

Figure S21. Evolution of Mean Square Displacement (MSD) during NVT-MD ReaxFF simulations of Pure Bulk LiF (LiF) and point defects: vacancy ($Li_{0.9}F$) and interstitial ($Li_{1.1}F$) at 500 K. The markers (square, circle, and triangular) represent the corresponding systems in the plot. The simulations were performed using ReaxFF parameterizations by Yun et al.[7] (blue), Wang et al.[8] (green), and our proposed new parameterization (red). The dashed line indicates the linear model ($MSD = 6D \cdot t$) employed to compute diffusivity in each simulation.

5 Reparameterized ReaxFF

```
! ReaxFF force field C/H/O/Si/Li/F by P. De Angelis (reparameterized from ReaxFF  by Yun, Kang-Seop, et al. (2017).).
 39       ! Number of general parameters
   50.0000 !Overcoordination parameter
    9.5469 !Overcoordination parameter
   26.5405 !Valency angle conjugation parameter
    1.7224 !Triple bond stabilisation parameter
    6.8702 !Triple bond stabilisation parameter
   60.4850 !C2-correction
    1.0588 !Undercoordination parameter
    4.6000 !Triple bond stabilisation parameter
   12.1176 !Undercoordination parameter
   13.3056 !Undercoordination parameter
  -70.5044 !Triple bond stabilization energy
    0.0000 !Lower Taper-radius
   10.0000 !Upper Taper-radius
    2.8793 !Not used
   33.8667 !Valency undercoordination
    6.0891 !Valency angle/lone pair parameter
    1.0563 !Valency angle
    2.0384 !Valency angle parameter
    6.1431 !Not used
    6.9290 !Double bond/angle parameter
    0.3989 !Double bond/angle parameter: overcoord
    3.9954 !Double bond/angle parameter: overcoord
   -2.4837 !Not used
    5.7796 !Torsion/BO parameter
   10.0000 !Torsion overcoordination
    1.9487 !Torsion overcoordination
   -1.2327 !Conjugation 0 (not used)
    2.1645 !Conjugation
    1.5591 !vdWaals shielding
    0.0010 !Cutoff for bond order (*100)
    2.1365 !Valency angle conjugation parameter
    0.6991 !Overcoordination parameter
   50.0000 !Overcoordination parameter
    1.8512 !Valency/lone pair parameter
    0.5000 !Not used
   20.0000 !Not used
    5.0000 !Molecular energy (not used)
    0.0000 !Molecular energy (not used)
    2.6962 !Valency angle conjugation parameter
 6      ! Nr of atoms; cov.r; valency; a.m.; Rvdw; Evdw; gammaEEM; cov.r2; #el.
        ! alfa; gammavdW; valency13; Eunder; Eover; chiEEm; etaEEM; n.u1
        ! cov.r3; Elp; Heat inc.; 13BO1; 13BO2; 13BO3; n.u2; n.u3
        ! ov/un; vval1;  vval2;  vval3;  vval4;  n.u5; n.u6; n.u7
 C    1.3825   4.0000  12.0000   1.9133   0.1853   0.9000   1.1359   4.0000
      9.7602   2.1346   4.0000  33.2433  79.5548   5.8678   7.0000   0.0000
      1.2104   0.0000 199.0303   8.6991  34.7289  13.3894   0.8563   0.0000
     -2.8983   2.5000   1.0564   4.0000   2.9663   0.0000   0.0000   0.0000
 H    0.7853   1.0000   1.0080   1.5904   0.0419   1.0206  -0.1000   1.0000
      9.3557   5.0518   1.0000   0.0000 121.1250   5.3200   7.4366   1.0000
     -0.1000   0.0000  62.4879   1.9771   3.3517   0.7571   1.0698   0.0000
    -15.7683   2.1488   1.0338   1.0000   2.8793   0.0000   0.0000   0.0000
 O    1.2450   2.0000  15.9990   2.3890   0.1000   1.0898   1.0548   6.0000
      9.7300  13.8449   4.0000  37.5000 116.0768   8.5000   8.3122   2.0000
      0.9049   0.4056  59.0626   3.5027   0.7640   0.0021   0.9745   0.0000
     -3.5500   2.9000   1.0493   4.0000   2.9225   1.3000   0.2000  13.0000
```

```
Si   2.2902   4.0000  28.0600   1.8354   0.2110   0.5947   1.2962   4.0000
    11.1336   3.1831   4.0000  21.7115 139.9309   4.2033   5.5558   0.0000
    -1.0000   0.0000 104.0000   9.0751  23.8188   0.8381   0.8563   0.0000
    -4.1684   2.0754   1.0338   4.0000   2.5791   1.4000   0.2000  13.0000
Li   1.9205   1.0000   6.9410   1.8896   0.0905   0.4668  -0.1000   1.0000
     9.9084   1.0896   1.0000   0.0000   0.0000  -6.4188  15.0000   0.0000
    -1.0000   0.0000  37.5000   5.4409   6.9107   0.1973   0.8563   0.0000
   -25.0000   2.2989   1.0338   1.0000   2.8103   1.3000   0.2000  13.0000
F    1.2705   1.0000  18.9984   1.4100   0.0442   0.0135  -0.1000   7.0000
    11.6107   5.4481   4.0000  62.1473   0.1252  15.4329  17.3228   0.0000
    -1.0000  35.0000   1.5000   6.9821   4.1799   1.0561   0.0000   0.0000
    -2.2869   2.7340   1.0493   4.0000   3.0013   0.0000   0.0000   0.0000
21    ! Nr of bonds; Edis1; Edis2; Edis3; pbe1;  pbo5; 13corr; pbo6; kov
      ! pbe2; pbo3;  pbo4; n.u1; pbo1; pbo2; ovcorr; n.u2
  1  1 156.5953 100.0397  80.0000  -0.8157  -0.4591   1.0000  37.7369   0.4235
         0.4527  -0.1000   9.2605   1.0000  -0.0750   6.8316   1.0000   0.0000
  1  2 170.2316   0.0000   0.0000  -0.5931   0.0000   1.0000   6.0000   0.7140
         5.2267   1.0000   0.0000   1.0000  -0.0500   6.8315   0.0000   0.0000
  1  4  94.5912  50.1197   0.0000  -0.5712  -0.5558   1.0000  17.2117   0.0308
         2.3951  -1.1892   8.6403   1.0000  -0.1028   5.4278   1.0000   0.0000
  2  2 156.0973   0.0000   0.0000  -0.1377   0.0000   1.0000   6.0000   0.8240
         2.9907   1.0000   0.0000   1.0000  -0.0593   4.8358   0.0000   0.0000
  1  3 224.8293  31.8847  89.5456  -1.4925  -0.0850   1.0000  13.4838   1.1008
         0.7387  -0.7228   5.1953   1.0000  -0.1175   6.4319   0.0000   0.0000
  3  3 142.2858 145.0000  50.8293   0.2506  -0.1000   1.0000  29.7503   0.6051
         0.3451  -0.1055   9.0000   1.0000  -0.1225   5.5000   1.0000   0.0000
  2  3 160.0000   0.0000   0.0000  -0.5725   0.0000   1.0000   6.0000   0.5626
         1.1150   1.0000   0.0000   0.0000  -0.0920   4.2790   0.0000   0.0000
  2  4 101.1840   0.0000   0.0000  -0.1751   0.0000   1.0000   6.0000   1.1044
         7.3549   1.0000   0.0000   1.0000  -0.0450   7.9080   0.0000   0.0000
  3  4 274.8339   5.0000   0.0000  -0.5884  -0.3000   1.0000  36.0000   0.2131
         9.9772  -0.2572  28.8153   1.0000  -0.1130   8.4790   6.0658   0.0000
  4  4  61.1127  85.8146  30.0000  -0.8197  -0.3000   1.0000  16.0000   0.1386
         0.1307  -0.8055   7.1248   1.0000  -0.0674   8.2374   0.0000   0.0000
  1  5  10.0540   0.0000   0.0000   0.3005  -0.3000   0.0000   6.0000   0.2953
         0.2679  -0.2534  12.0019   1.0000  -0.1143   7.5211   0.0000   0.0000
  2  5  63.4649   0.0000   0.0000   0.0294   0.0000   0.0000   6.0000   0.4868
         0.3090   0.0000  12.0000   1.0000  -0.0800   5.1033   0.0000   0.0000
  5  3  78.3666  -0.0200   0.0000  -1.0000   0.3000   0.0000   6.0000   0.3228
         0.2022  -0.2500  11.9965   1.0000  -0.1276   7.8656   0.0000   0.0000
  5  4  23.1963   0.0000   0.0000   1.0000   0.3000   0.0000  26.0000   0.5185
         0.0812   0.0000  12.0000   1.0000  -0.1142   6.0525   0.0000   0.0000
  5  5  16.7443   0.0000   0.0000   1.0420   0.3000   0.0000  26.0659   0.5820
         1.6137  -0.0805  12.0000   1.0000  -0.1646   4.7897   0.0000   0.0000
  1  6 166.8800   0.0000   0.0000   0.4753  -0.5000   1.0000  35.0000   1.3151
         3.1303  -0.2500  15.0000   1.0000  -0.9475   7.1188   1.0000   0.0000
  6  2 260.6892   0.0000   0.0000  -0.6690   0.0000   1.0000   6.0000   2.3211
         8.8937   1.0000   0.0000   1.0000  -0.3966   9.2031   0.0000   0.0000
  6  3  99.9065   0.0000   0.0000  -1.0000   0.0000   1.0000   6.0000   0.7194
         0.5928   1.0000   0.0000   1.0000  -0.1318   8.4278   0.0000   0.0000
  6  6  65.8563   0.0000   0.0000  -0.0630  -0.5267   1.0116  30.2616   0.2245
        -0.2306  -0.2161  15.3928   1.0000  -0.1007   8.6628   0.0597   0.0000
  6  5  24.7086   0.0000   0.0000  -1.3775  -0.0961   0.4644  45.9272   0.6224
         6.9172  -0.0851  14.8066   1.0000  -0.0752   9.0692   0.7043   0.0000
  6  4 284.8610   0.0000   0.0000  -0.8680  -0.5000   1.0000  35.0000   1.4117
         3.5449  -0.2500  15.0000   1.0000  -0.1198   6.0380   1.0000   0.0000
15    ! Nr of off-diagonal terms; Ediss; Rvdw; alfa; cov.r; cov.r2; cov.r3
  1  2   0.1219   1.4000   9.8442   1.1203  -1.0000  -1.0000
  1  3   0.1893   1.7076  10.2970   1.3608   1.0384   1.0646
  1  4   0.5876   1.3349  13.4198   1.4988   1.6946  -1.0000
```

```
  2  3   0.0283   1.2885  10.9190   0.9215  -1.0000  -1.0000
  2  4   0.1035   1.3327  11.5963   1.3977  -1.0000  -1.0000
  3  4   0.1836   1.9157  10.9070   1.7073   1.2375  -1.0000
  1  5   0.0610   1.5665  11.4404   1.2079  -1.0000  -1.0000
  2  5   0.2966   1.2550  10.2920   1.1989  -1.0000  -1.0000
  5  3   0.0790   2.2000   9.0491   1.8165  -1.0000   1.0000
  5  4   0.1271   2.0090  11.5659   1.7156   1.0000   1.0000
  1  6   0.0749   1.7702  12.0430   1.6315  -1.0000  -1.0000
  6  2   0.0702   1.1145  12.1833   1.0722  -1.0000  -1.0000
  6  3   0.1293   1.3773  11.4683   1.3410  -1.0000  -1.0000
  6  5   0.0495   1.2573  15.6983   0.9415  -1.0000  -1.0000
  6  4   0.0996   1.5863  13.3401   1.5077  -1.0000  -1.0000
 52    ! Nr of angles; Theta0; ka; kb; pconj; pv2; kpenal; pv3
  1  1  1  67.2326  22.0695   1.6286   0.0000   1.7959  15.4141   1.8089
  1  1  2  65.2527  14.3185   6.2977   0.0000   0.5645   0.0000   1.1530
  2  1  2  70.0840  25.3540   3.4508   0.0000   0.0050   0.0000   3.0000
  1  2  2   0.0000   0.0000   6.0000   0.0000   0.0000   0.0000   1.0400
  1  2  1   0.0000   3.4110   7.7350   0.0000   0.0000   0.0000   1.0400
  2  2  2   0.0000  27.9213   5.8635   0.0000   0.0000   0.0000   1.0400
  1  1  3  94.2903  59.3577   5.0000   0.0000   1.8747   1.0000   1.3538
  3  1  3  86.8971  23.9117   5.0000   0.0000   0.0499   1.0000   2.8723
  1  3  1  49.8324  39.3271   5.0000   0.0000   3.0156   0.0000   2.7534
  1  3  2  70.1101  13.1217   4.4734   0.0000   0.8433   0.0000   3.0000
  1  3  3  81.9029  32.2258   1.7397   0.0000   0.9888  68.1072   1.7777
  1  2  3   0.0000  25.0000   3.0000   0.0000   1.0000   0.0000   1.0400
  3  3  3  80.7324  30.4554   0.9953   0.0000   1.6310  50.0000   1.0783
  2  3  3  75.6935  50.0000   2.0000   0.0000   1.0000   0.0000   1.1680
  2  3  2  85.8000   9.8453   2.2720   0.0000   2.8635   0.0000   1.5800
  3  2  3   0.0000  15.0000   2.8900   0.0000   0.0000   0.0000   2.8774
  2  2  3   0.0000   8.5744   3.0000   0.0000   0.0000   0.0000   1.0421
  4  4  4  66.2921  19.5195   0.9624   0.0000   0.1000   0.0000   1.2636
  2  4  4  68.5501  19.4239   2.3592   0.0000   0.2029   0.0000   1.0000
  2  4  2  70.7499  11.4850   4.6606   0.0000   1.5647   0.0000   1.0902
  3  4  4  86.3294  18.3879   5.8529   0.0000   1.7361   0.0000   1.2310
  2  4  3  73.6998  40.0000   1.8782   0.0000   4.0000   0.0000   1.1290
  3  4  3  79.5581  34.9140   1.0801   0.0000   0.1632   0.0000   2.2206
  4  3  4  82.3364   4.7350   1.3544   0.0000   1.4627   0.0000   1.0400
  2  3  4  90.0000   6.6857   1.6689   0.0000   2.5771   0.0000   1.0400
  3  3  4  92.1207  24.3937   0.5000   0.0000   1.7208   0.0000   3.0000
  2  2  4   0.0000   0.0100   1.0000   0.0000   1.0000   0.0000   2.0000
  4  2  4   0.0000   4.4216   0.8596   0.0000   0.9624   0.0000   1.0000
  3  2  4   0.0000   5.0000   1.0000   0.0000   1.0000   0.0000   1.2500
  3  5  3  60.0000   0.0000   1.0000   0.0000   1.0000   0.0000   1.0000
  5  3  3  81.6233  30.0000   2.0000   0.0000   1.0000   0.0000   1.0000
  5  3  5  67.5247   6.4512   4.0000   0.0000   1.0000   0.0000   2.8079
  5  3  4  62.6634   8.4441   2.5120   0.0000   1.0000   0.0000   1.0000
  1  5  3   0.0000   0.0100   1.0000   0.0000   1.0000   0.0000   1.0000
  1  3  5  98.9874   6.7756   0.2680   0.0000   3.8836   0.0000   1.0000
  5  1  3  99.6399   0.0100   3.8420   0.0000   2.0653   0.0000   1.8902
  1  1  4  70.8533  23.2816   2.7470   0.0000   2.0166   0.0000   2.2246
  1  4  1  69.9335  20.9406   1.8375   0.0000   0.2981   0.0000   2.1195
  4  1  4  50.9317  18.9333   1.8833   0.0000   0.2981   0.0000   2.3646
  1  4  4  69.3369  19.6964   2.0703   0.0000   1.0031   0.0000   1.0400
  2  1  4  72.5949  13.8347   2.4952   0.0000   1.0000   0.0000   1.0400
  1  4  2  72.5949  14.8347   2.4952   0.0000   1.0000   0.0000   1.0400
  1  2  4   0.0000   2.5000   1.0000   0.0000   1.0000   0.0000   1.2500
  1  1  6  35.0676  44.0627   0.4803   0.0000   0.1357   0.0000   0.6875
  6  1  6  90.2886  41.7871   0.4774   0.0000   0.2704   0.0000   0.6548
  1  6  1  82.4766  33.5783   0.1356   0.0000   0.2024   0.0000   0.9612
  1  6  6   2.0340  25.1860   1.0950   0.0000   0.1315   0.0000   0.9638
```

```
 5  6  5  75.9308  39.5963   1.5406   0.0000   5.0016   0.0000   1.2226
 6  5  6  85.2677  38.4940   1.6737   0.0000   5.0138   0.0000   1.3107
 6  3  6  86.4972  15.1409   1.1470   0.0000   0.2045   0.0000   2.4012
 6  3  3  73.0506  30.3751   2.0217   0.0000   1.7756   0.0000   2.9301
 6  4  6  79.1640  38.5410   1.3735   0.0000   4.9517   0.0000   2.2216
34     ! Nr of torsions; V1; V2; V3; V2(BO); vconj; n.u1; n.u2
 1  1  1  1  -0.2500  11.5822   0.1899  -4.7057  -2.2047   0.0000   0.0000
 1  1  1  2  -0.2500  31.2596   0.1709  -4.6391  -1.9002   0.0000   0.0000
 2  1  1  2  -0.1770  30.0252   0.4340  -5.0019  -2.0697   0.0000   0.0000
 1  1  1  3  -0.5000   5.0000  -0.5000  -9.0000  -1.0000   0.0000   0.0000
 2  1  1  3  -0.5000   8.1082   0.1710  -8.1074  -1.0000   0.0000   0.0000
 3  1  1  3  -1.4477  16.6853   0.6461  -4.9622  -1.0000   0.0000   0.0000
 1  1  3  1  -0.2300  46.8253  -0.2848  -2.6326  -1.0000   0.0000   0.0000
 1  1  3  2   1.2044  80.0000  -0.3139  -6.1481  -1.0000   0.0000   0.0000
 2  1  3  1  -2.5000  31.0191   0.6165  -2.7733  -2.9807   0.0000   0.0000
 2  1  3  2  -2.4875  70.8145   0.7582  -4.2274  -3.0000   0.0000   0.0000
 1  1  3  3  -0.0002  20.1851   0.1601  -9.0000  -2.0000   0.0000   0.0000
 2  1  3  3  -1.4383  80.0000   1.0000  -3.6877  -2.8000   0.0000   0.0000
 3  1  3  1  -1.2244  77.8133  -0.4738  -4.7499  -3.0000   0.0000   0.0000
 3  1  3  2  -2.5000  70.3345  -1.0000  -5.5315  -3.0000   0.0000   0.0000
 3  1  3  3  -0.1583  20.0000   1.5000  -9.0000  -2.0000   0.0000   0.0000
 1  3  3  1   0.0002  80.0000  -1.5000  -4.4848  -2.0000   0.0000   0.0000
 1  3  3  2  -2.1289  12.8382   1.0000  -5.6657  -2.9759   0.0000   0.0000
 1  3  3  3   2.5000 -25.0000   1.0000  -2.5000  -1.0000   0.0000   0.0000
 2  3  3  3   0.8302  -4.0000  -0.7763  -2.5000  -1.0000   0.0000   0.0000
 3  3  3  3  -2.5000  -4.0000   1.0000  -2.5000  -1.0000   0.0000   0.0000
 0  1  1  0   0.0000   0.0000   0.0000   0.0000   0.0000   0.0000   0.0000
 0  1  2  0   0.0000   0.0000   0.0000   0.0000   0.0000   0.0000   0.0000
 0  2  2  0   0.0000   0.0000   0.0000   0.0000   0.0000   0.0000   0.0000
 0  2  3  0   0.0000   0.1000   0.0200  -2.5415   0.0000   0.0000   0.0000
 0  3  3  0   0.5511  25.4150   1.1330  -5.1903  -1.0000   0.0000   0.0000
 2  4  4  2   0.0000   0.0000   0.0640  -2.4426   0.0000   0.0000   0.0000
 2  4  4  4   0.0000   0.0000   0.1587  -2.4426   0.0000   0.0000   0.0000
 0  2  4  0   0.0000   0.0000   0.1200  -2.4847   0.0000   0.0000   0.0000
 1  1  1  6   0.0000  42.3172   3.4546 -22.6501  -1.7255   0.0000   0.0000
 6  1  1  6   0.0000  75.5402  -0.7497  -4.0257  -1.7255   0.0000   0.0000
 0  1  6  0   4.0000  45.8264   0.9000  -4.0000   0.0000   0.0000   0.0000
 0  6  6  0   4.0000  45.8264   0.9000  -4.0000   0.0000   0.0000   0.0000
 0  5  6  0   0.0336   0.6333  -0.0621  -0.0007  -0.0578   0.0000   0.0000
 0  4  6  0   4.7334  44.7693   1.2361  -4.7157   0.0000   0.0000   0.0000
2      ! Nr of hydrogen bonds; Rhb; Dehb; vhb1; vhb2
 3  2  3   2.1200  -3.5800   1.4500  19.5000
 6  2  3   2.1200  -2.0000   1.4500  19.5000
```

References

1. Wilkinson, M. D. *et al.* The fair guiding principles for scientific data management and stewardship. *Sci. data* **3**, 1–9, DOI: https://doi.org/10.1038/sdata.2016.18 (2016).

2. Winter, M. The solid electrolyte interphase–the most important and the least understood solid electrolyte in rechargeable li batteries. *Zeitschrift für physikalische Chemie* **223**, 1395–1406, DOI: https://doi.org/10.1524/zpch.2009.6086 (2009).

3. Van Duin, A. C., Dasgupta, S., Lorant, F. & Goddard, W. A. Reaxff: a reactive force field for hydrocarbons. *The J. Phys. Chem. A* **105**, 9396–9409, DOI: https://doi.org/10.1021/jp004368u (2001).

4. Amsterdam Modeling Suite, SCM website.

5. Komissarov, L., Ruger, R., Hellstrom, M. & Verstraelen, T. Params: parameter optimization for atomistic and molecular simulations. *J. Chem. Inf. Model.* **61**, 3737–3743, DOI: https://doi.org/10.1021/acs.jcim.1c00333 (2021).

6. Celik, F. A. & Karabulut, E. A new design of borax-cannabinol nanomaterial used to strengthen concrete structures: Non-scc-gfn1xtb model. *Solid State Commun.* **358**, 114995, DOI: https://doi.org/10.1016/j.ssc.2022.114995 (2022).

7. Yun, K.-S. *et al.* Simulation protocol for prediction of a solid-electrolyte interphase on the silicon-based anodes of a lithium-ion battery: Reaxff reactive force field. *The J. Phys. Chem. Lett.* **8**, 2812–2818, DOI: https://doi.org/10.1021/acs.jpclett.7b00898 (2017).

8. Wang, Y., Liu, Y., Tu, Y. & Wang, Q. Reductive decomposition of solvents and additives toward solid-electrolyte interphase formation in lithium-ion battery. *The J. Phys. Chem. C* **124**, 9099–9108, DOI: https://doi.org/10.1021/acs.jpcc.9b10535 (2020).

9. Jain, A. *et al.* Commentary: The materials project: A materials genome approach to accelerating materials innovation. *APL materials* **1**, 011002, DOI: https://doi.org/10.1063/1.4812323 (2013).
